# Supplementary material for: Autonomous error correction of a single logical qubit using two transmons
Source: Nat Commun. 2024 Feb 23;15:1681. doi: 10.1038/s41467-024-45858-z (PMC10891116; doi:10.1038/s41467-024-45858-z)
Supplement: Supplementary file 1 — Supplementary Information [file 41467_2024_45858_MOESM1_ESM.pdf]

Autonomous error correction of a single logical qubit using two transmons

SUPPLEMENTARY NOTE 1: DEVICE PARAMETERS

Transition ($\Phi_{\text{DC}} = 0$)	T_1 (μs)	T_R (μs)	T_{echo} (μs)
$Q_1 \lvert e\rangle \to \lvert g\rangle$	31.6	28.4	26.6
$Q_2 \lvert e\rangle \to \lvert g\rangle$	2.8	4.9	-
**Transition ($\Phi_{\text{DC}} = 0.3795\Phi_0$)**	**T_1 (μs)**	**T_R (μs)**	**T_{echo} (μs)**
$Q_1 \lvert e\rangle \to \lvert g\rangle$	24.3	15.2	24.6
$Q_2 \lvert e\rangle \to \lvert g\rangle$	9.1	9.8	14.3
$Q_1 \lvert f\rangle \to \lvert e\rangle$	27.1	16.7	29.3
$Q_2 \lvert f\rangle \to \lvert e\rangle$	26.7	20.1	34.3
$R_1 \lvert 1\rangle \to \lvert 0\rangle$	0.3		
$R_2 \lvert 1\rangle \to \lvert 0\rangle$	0.3		

Supplementary Table 1. Device coherence parameters at two DC flux points. Q_2 has lower coherence at the zero flux point because of the presence of a two-level system (TLS).

Supplementary Fig. 1a shows a false-colored optical image of the device. Relevant coherence parameters and frequencies at the operating point (coupler DC flux bias $\Phi_{\text{DC}} = 0.3795\Phi_0$) without external drives are listed in Supplementary Table 1 and Supplementary Table 2. The ZZ coupling (dispersive shifts) between two-transmon energy levels are measured in the experiment through Ramsey fringe frequency difference (Supplementary Note 7), and the cross-Kerr couplings J_{11}, J_{21}, J_{12}, J_{22} are calculated from those measurement results. In the experiment, we separately measure all 7 ZZs through Ramsey frequency difference (See Supplementary Fig. 11). The four independent J_{jk} are inferred from the experimentally measured seven cross-Kerr values: we choose the J_{jk} set that minimizes the Euclidean distance between the seven experimental and the theoretical ZZ values calculated from Supplementary Eq. (1).

Parameter	Symbol	Value/2π
Q_1 ge frequency	ω_{q1}	3.2049 GHz
Q_2 ge frequency	ω_{q2}	3.6625 GHz
Q_1 anharmonicity	α_1	−116.4 MHz
Q_2 anharmonicity	α_2	−159.6 MHz
R_1 frequency	ω_{r1}	4.9946 GHz
R_2 frequency	ω_{r2}	5.4505 GHz
R_1 dispersive shift	χ_1	−180 kHz
R_2 dispersive shift	χ_2	−330 kHz
$(E_{\lvert ee\rangle} - E_{\lvert ge\rangle}) - (E_{\lvert eg\rangle} - E_{\lvert gg\rangle})$	ZZ_{ge}	−261 kHz
$(E_{\lvert fe\rangle} - E_{\lvert ee\rangle}) - (E_{\lvert fg\rangle} - E_{\lvert eg\rangle})$	ZZ_{ef1}	−130 kHz
$(E_{\lvert ef\rangle} - E_{\lvert ee\rangle}) - (E_{\lvert gf\rangle} - E_{\lvert ge\rangle})$	ZZ_{ef2}	−301 kHz
$(E_{\lvert ff\rangle} - E_{\lvert ef\rangle}) - (E_{\lvert fg\rangle} - E_{\lvert eg\rangle})$	ZZ_{ff1}	−171 kHz
$(E_{\lvert ff\rangle} - E_{\lvert fe\rangle}) - (E_{\lvert gf\rangle} - E_{\lvert ge\rangle})$	ZZ_{ff2}	−289 kHz
$(E_{\lvert ef\rangle} - E_{\lvert gf\rangle}) - (E_{\lvert eg\rangle} - E_{\lvert gg\rangle})$	ZZ_{gf1}	−619 kHz
$(E_{\lvert fe\rangle} - E_{\lvert fg\rangle}) - (E_{\lvert ge\rangle} - E_{\lvert gg\rangle})$	ZZ_{gf2}	−464 kHz
Coefficient of $n_{q1}n_{q2}$	J_{11}	−312 kHz
Coefficient of $n_{q1}^2 n_{q2}$	J_{21}	25 kHz
Coefficient of $n_{q1}n_{q2}^2$	J_{12}	−49 kHz
Coefficient of $n_{q1}^2 n_{q2}^2$	J_{22}	43 kHz

Supplementary Table 2. Device frequencies in the absence of external drives. J_{jk} are inferred from the 7 ZZ values measured in the experiment.

Capacitance	(fF)	Josephson Energy	(GHz)
C_{q1}	165.9	E_{j1}	12.4
C_{q2}	123.4	E_{j2}	12.1
C_c	178.3	E_{jc}	1106.0
C_{q12}	2.0		

Supplementary Table 3. Capacitances and Josephson energies used in the quantization. Capacitances are extracted through finite-element simulations in ANSYS Q3D, and Josephson energies are calculated from the room-temperature resistances of nominally identical test junctions on the same chip.

$$\begin{cases} ZZ_{ge} &= J_{11} + J_{12} + J_{21} + J_{22} \\ ZZ_{ef1} &= J_{11} + 3J_{12} + J_{21} + 3J_{22} \\ ZZ_{ef2} &= J_{11} + J_{12} + 3J_{21} + 3J_{22} \\ ZZ_{ff1} &= 2J_{11} + 6J_{12} + 4J_{21} + 12J_{22} \\ ZZ_{ff2} &= 2J_{11} + 4J_{12} + 6J_{21} + 12J_{22} \\ ZZ_{gf1} &= 2J_{11} + 2J_{12} + 4J_{21} + 4J_{22} \\ ZZ_{gf2} &= 2J_{11} + 4J_{12} + 2J_{21} + 4J_{22} \end{cases} \tag{1}$$

SUPPLEMENTARY NOTE 2: CIRCUIT HAMILTONIAN AND SIDEBAND STRENGTH

We first consider the Hamiltonian of the two transmons:

$$\begin{aligned} H_Q = &\vec{n}^{\intercal} C_L^{-1} \vec{n} - E_{j1}\cos(\varphi_c - \varphi_1) - E_{j2}\cos(\varphi_2 - \varphi_c) \\ &- E_{jc}\cos\left(\pi\frac{\Phi_{\text{ext}}}{\Phi_0}\right)\cos(\varphi_c), \end{aligned} \tag{2}$$

$$C_L = \begin{bmatrix} C_{q1} + C_{q12} & -C_{q12} & 0 \\ -C_{q12} & C_{q2} + C_{q12} & 0 \\ 0 & 0 & C_{q1} + C_{q2} + C_{qc} \end{bmatrix}, \tag{3}$$

$$\vec{n}^{\intercal} = (n_1, n_2, n_c), \; [n_j, \varphi_j] = -i. \tag{4}$$

Here $\vec{n}$ and $\vec{\varphi}$ are the charge and phase variables and can be found through the Legendre transformation. Supplementary Table 3 includes all coefficients used in the quantization. Then we extract the linear part of H_Q to obtain

$$\begin{aligned} H_0 = \vec{n}^{\intercal} C_L^{-1} \vec{n} + \frac{E_{j1}}{2}(\varphi_c - \varphi_1)^2 + \frac{E_{j2}}{2}(\varphi_2 - \varphi_c)^2 \\ + \frac{E_{jc}}{2}\cos\left(\pi\frac{\Phi_{\text{ext}}}{\Phi_0}\right)\varphi_c^2. \end{aligned} \tag{5}$$

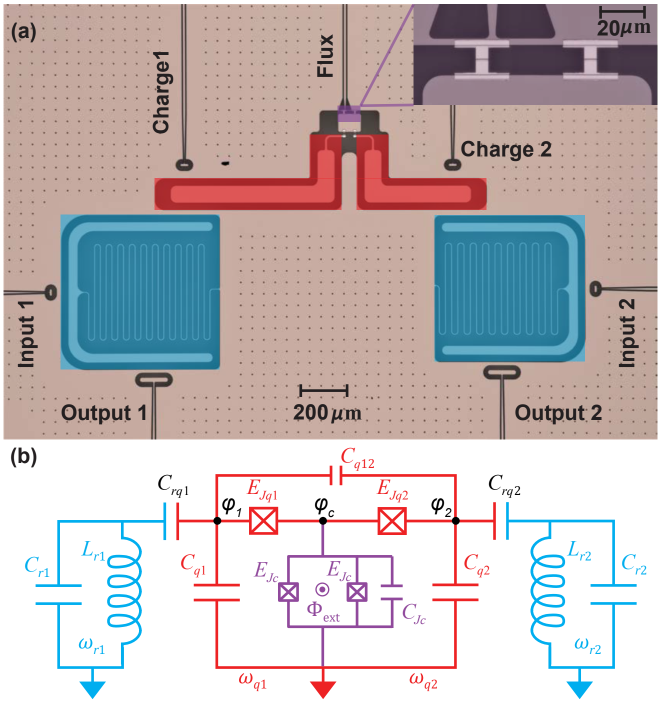

Supplementary Figure 1. The device. (a) False-colored optical image. Two transmons (red) are inductively connected through a SQUID loop (purple, inset shows zoomed-in image). An on-chip flux line is coupled to the SQUID for activating QQ sidebands through parametric RF flux modulation at the proper DC flux position. Each transmon is capacitively coupled to the readout resonator (blue). Single transmon pulses are sent through the resonator input lines. QR sidebands are applied through corresponding charge lines. (b) Circuit schematic diagram.

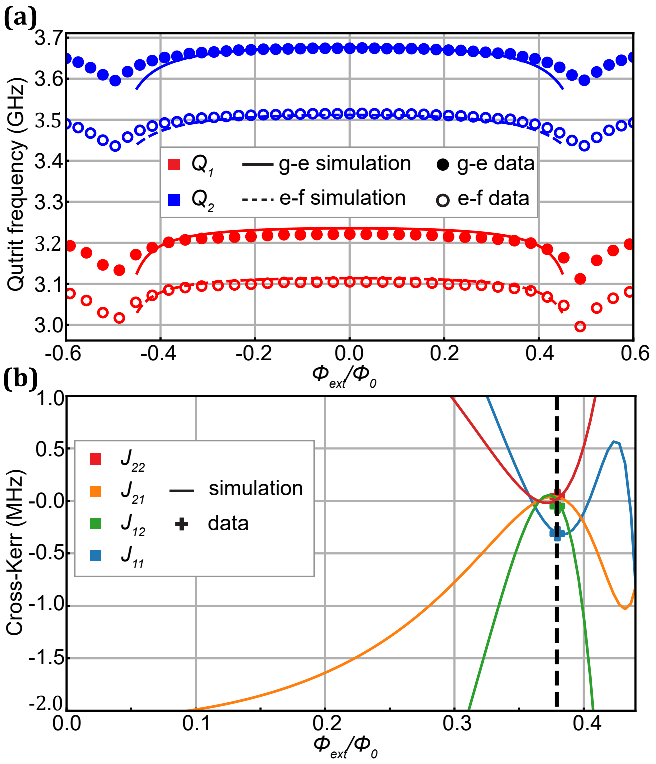

Supplementary Figure 2. Circuit quantization results of H_Q. Comparison of (a) transmon frequencies and (b) cross-Kerr couplings between simulation and experiment. Q1 (red) and Q2's (blue) $|g\rangle \leftrightarrow |e\rangle$ and $|e\rangle \leftrightarrow |f\rangle$ frequencies from numerical calculation and experiment are plotted as a function of Φ_{ext}. Four inter-qutrit cross-Kerr coupling strengths, J_{11}, J_{21}, J_{12} and J_{22} are calculated, and experiment data are marked out on the Star code operating point (dashed line).

Next, we rewrite the charge and phase variables in the dressed basis with the unitary transformation matrix U such that H_0 is simultaneously diagonalized to find out the normal modes,

$$H_0 = \sum_{j=1,2,c} \left(\tilde{C}_j \tilde{n}_j^2 + \tilde{D}_j {\tilde{\varphi}_j}^2 \right), \tag{6}$$

$$\overrightarrow{\tilde{n}} = (\tilde{n}_1, \tilde{n}_2, \tilde{n}_c)^{\intercal} = U^{-1}\overrightarrow{n}, \tag{7}$$

$$\overrightarrow{\tilde{\varphi}} = (\tilde{\varphi}_1, \tilde{\varphi}_2, \tilde{\varphi}_c)^{\intercal} = U^{-1}\overrightarrow{\varphi}, \tag{8}$$

$$U = \begin{bmatrix} U_{11} & U_{12} & U_{1c} \\ U_{21} & U_{22} & U_{2c} \\ U_{c1} & U_{c2} & U_{cc} \end{bmatrix}. \tag{9}$$

In the dressed basis, the nonlinear part is reintroduced in the Hamiltonian to get

$$\begin{aligned} H_Q =& \sum_{j=1,2,c} \left(\tilde{C}_j \tilde{n}_j^2 \right) \\ &- E_{j1} \cos\left(\sum_{j=1,2,c} (U_{cj}\tilde{\varphi}_j - U_{1j}\tilde{\varphi}_j) \right) \\ &- E_{j2} \cos\left(\sum_{j=1,2,c} (U_{2j}\tilde{\varphi}_j - U_{cj}\tilde{\varphi}_j) \right) \\ &- E_{jc} \cos\left(\pi \frac{\Phi_{\text{ext}}}{\Phi_0} \right) \cos\left(\sum_{j=1,2,c} U_{cj}\tilde{\varphi}_j \right), \end{aligned} \tag{10}$$

with

$$\tilde{n}_j = \frac{i}{\sqrt{2}} \sqrt{\frac{\tilde{D}_j}{\tilde{C}_j}} \left(a_{qj}^{\dagger} - a_{qj} \right), \tag{11}$$

$$\tilde{\varphi}_j = \frac{1}{\sqrt{2}} \sqrt{\frac{\tilde{C}_j}{\tilde{D}_j}} \left(a_{qj}^{\dagger} + a_{qj} \right). \tag{12}$$

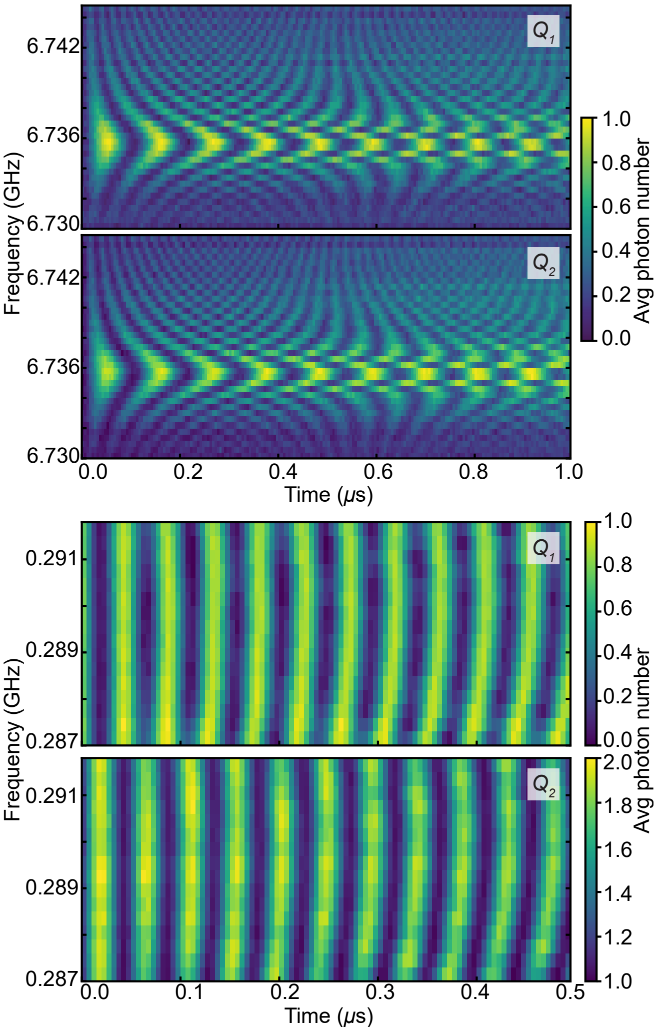

Supplementary Figure 3. Chevron plots for fast QQ sidebands. The state of both transmons are simultaneously read out and shown as photon numbers. Top two figures show 9 MHz $|gg\rangle \leftrightarrow |ee\rangle$, and bottom two demonstrate 21 MHz $|ee\rangle \leftrightarrow |gf\rangle$ oscillations.

We use the scQubits package [1] to quantize the Hamiltonian. The comparison between numerical values and experimental data are shown in Supplementary Fig. 2. When Φ_{ext} is biased close to $\Phi_0/2$, deviation appeared in numerics. This comes from the asymmetry of SQUID junctions' resistances and parasitic SQUID loop inductance. This region of deviation is far from the experimental bias point. Around the DC flux position where the Star code protocol is implemented (marked as Supplementary Fig. 2b dash line), there is a good agreement between simulated and experimental values for both transmons' frequencies and cross-Kerr couplings.

The QQ sidebands are realized through parametric RF flux modulation of the coupler. To understand the sideband rate, we follow the previous paper[2] and apply an adiabatic approximation to the Hamiltonian: The coupler mode frequency remains high (> 15 GHz) above transmons' frequencies (< 4 GHz) in the system. Therefore the coupler can be assumed static at the ground state. The non-dynamical potential of the coupler mode is removed by minimizing the Hamiltonian. Transmons are treated as duffing oscillators when calculating the effective sideband rate. Keeping up to 2nd order expansions, the Hamiltonian H_{ad} under adiabatic approximation is

$$\begin{aligned} H_{\text{ad}} &= \omega_{q1} a_{q1}^\dagger a_{q1} + \omega_{q2} a_{q2}^\dagger a_{q2} \\ &+ \frac{\alpha_1}{2} a_{q1}^\dagger a_{q1}^\dagger a_{q1} a_{q1} + \frac{\alpha_2}{2} a_{q2}^\dagger a_{q2}^\dagger a_{q2} a_{q2} \\ &+ g_1(t)\left(a_{q1}^\dagger + a_{q1}\right)\left(a_{q2}^\dagger + a_{q2}\right) \\ &+ g_2\left(-a_{q1}^\dagger + a_{q1}\right)\left(-a_{q2}^\dagger + a_{q2}\right), \end{aligned} \tag{13}$$

$$g_1(t) = \frac{\sqrt{E_{j1}E_{j2}}}{2E_{jc}\cos\left(\pi\frac{\Phi_{\text{ext}}(t)}{\Phi_0}\right)}\sqrt{\omega_{q1}\omega_{q2}}, \tag{14}$$

$$g_2 = \frac{\sqrt{C_{q1}C_{q2}}}{2C_{q12}}\sqrt{\omega_{q1}\omega_{q2}}. \tag{15}$$

Here $g_1(t)$ and g_2 are flux-tunable inductive coupling strength and constant capacitive coupling strength. Plugging in the RF flux modulation $\frac{\pi\Phi_{\text{ext}}(t)}{\Phi_0} = \Phi_{\text{DC}} + \epsilon\cos(\omega_d t)$ into Supplementary Eq. (14) and assuming $\epsilon \ll \Phi_{\text{DC}}$, we obtain

$$\begin{aligned} g_1(t) &= \frac{\sqrt{E_{j1}E_{j2}}}{2E_{jc}}\sqrt{\omega_{q1}\omega_{q2}}\frac{1}{\cos(\Phi_{\text{DC}} + \epsilon\cos(\omega_d t))} \\ &= \frac{\sqrt{E_{j1}E_{j2}}}{2E_{jc}}\sqrt{\omega_{q1}\omega_{q2}}\frac{(1 + \epsilon\sin(\omega_d t)\tan(\Phi_{\text{DC}}))}{\cos(\Phi_{\text{DC}})}. \end{aligned} \tag{16}$$

Therefore the QQ sideband rate becomes (suppose $|\psi_1\rangle$ and $|\psi_2\rangle$ are states connected by the sideband)

$$\frac{\sqrt{E_{j1}E_{j2}}}{2E_{jc}}\sqrt{\omega_{q1}\omega_{q2}}\frac{\epsilon\tan(\Phi_{\text{DC}})}{\cos(\Phi_{\text{DC}})}A_{12}, \tag{17}$$

with

$$A_{12} = \langle\psi_1|\left(a_{q1}^\dagger + a_{q1}\right)\left(a_{q2}^\dagger + a_{q2}\right)|\psi_2\rangle, \tag{18}$$

and is proportional to the flux modulation rate. A_{12} is the state-dependent bosonic enhancement coefficient. Higher order corrections can be calculated using time-dependent Schrieffer–Wolff transformation[3], and for our inductive coupler, both QQ blue and red sideband will have a similar rate under the same ϵ.

Each transmon is capacitively coupled to the readout resonator, and the second-order QR error-correcting sidebands are generated through the charge drive at half the transition energy $\omega_{dqrj} = (\omega_{qj} + \omega_{rj} + \alpha_{qj})/2$ with drive

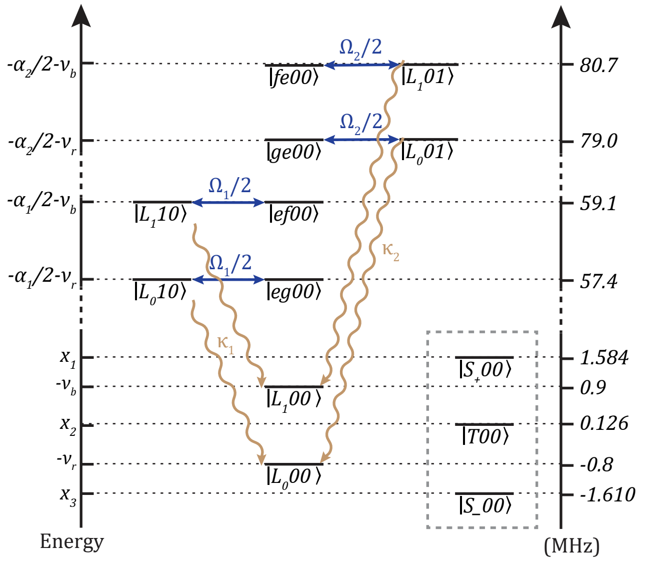

Supplementary Figure 4. Star code level diagram. The four QQ sidebands $|ee\rangle \leftrightarrow \{|gf\rangle, |fg\rangle, |gg\rangle, |ff\rangle\}$ create the level structure. The eigenstates without resonator photon population are grouped into three sets: Logical space $\{|L_0\rangle, |L_1\rangle\}$; error space $\{|eg\rangle, |ef\rangle, |ge\rangle, |fe\rangle\}$; quasi-stable space $\{|S_\pm\rangle, |T\rangle\}$. QR blue sidebands with rates Ω_1 and Ω_2 introduce the oscillation between the error states and the corresponding logical state with one photon in the resonator. The oscillation damps to the logical state quickly since resonator decay rate κ_j is large. $x_j, j = 1,2,3$ are the roots of Supplementary Eq. (37), which is related to both QQ sideband rate $\{W_r, W_b\}$ and detunings $\{\nu_r, \nu_b\}$. The right vertical axis shows the calculated energies of the relevant levels in the experiment.

amplitude ϵ_{qj},

$$\begin{aligned} H_{QRj} =& \omega_{qj} a_{qj}^\dagger a_{qj} + \frac{\alpha_j}{2}\left(a_{qj}^\dagger\right)^2 \left(a_{qj}\right)^2 + \omega_{rj} a_{rj}^\dagger a_{rj} \\ &+ g_{qrj}\left(-a_{qj}^\dagger + a_{qj}\right)\left(-a_{rj}^\dagger + a_{rj}\right) \\ &+ \epsilon_{qj}\left(a_{qj}^\dagger e^{-i\omega_{dqrj}t} + h.c.\right). \end{aligned} \tag{19}$$

The effective QR sideband rate is $\Omega_j = 16 g_{qrj}^3 \epsilon_{qj}^2 / (\omega_{qj} - \omega_{rj})^4$ [4]. Under the same Purcell limit $\left(\sim \frac{g_{qrj}^2 \kappa_j}{(\omega_{qj}-\omega_{rj})^2}\right)$ from the resonator, smaller QR frequency difference allows higher QR sideband rate for the same drive amplitude. In our experiment, the Purcell limit from the resonator is larger than 200 μs and does not limit the physical coherence time.

SUPPLEMENTARY NOTE 3: STAR CODE THEORY

A. Frame Transformation

The Star code requires the application of six always-on drives to a two-transmon-two-resonator system:

Two qubit-qubit (QQ) red sidebands $|ee\rangle \leftrightarrow \{|gf\rangle, |fg\rangle\}$ with rate W_r and detuning $\{\nu_r, -\nu_r\}$;

Two QQ blue sidebands $|ee\rangle \leftrightarrow \{|gg\rangle, |ff\rangle\}$ with rate W_b and detuning $\{-\nu_b, \nu_b\}$;

And two on-resonant qubit-resonator (QR) blue sidebands $|e0\rangle_j \leftrightarrow |f1\rangle_j, j = 1,2$ with rate Ω_j.

ZZ couplings between two transmons and between each transmon-resonator pair are first ignored for simplicity and can be reintroduced by shifting the diagonal energies. Their effects on the logical state coherence are discussed later. Under all the external drives, the lab frame Hamiltonian is

$$\begin{aligned} H_{\text{lab}} =& \sum_{j=1}^{2}\left(\omega_{qj} a_{qj}^\dagger a_{qj} + \frac{\alpha_j}{2} a_{qj}^\dagger a_{qj}^\dagger a_{qj} a_{qj} + \omega_{rj} a_{rj}^\dagger a_{rj}\right) \\ &+ H_{QQ} + \sum_{j=1}^{2} H_{QRj}, \end{aligned} \tag{20}$$

$$H_{QQ} = A_{QQ}(t)\left(a_{q1}^\dagger + a_{q1}\right)\left(a_{q2}^\dagger + a_{q2}\right), \tag{21}$$

$$H_{QRj} = A_{QRj}(t)\left(a_{qj}^\dagger + a_{qj}\right)\left(a_{rj}^\dagger + a_{rj}\right), \tag{22}$$

$$\begin{aligned} A_{QQ}(t) =& \frac{W_r}{\sqrt{2}} \cos\left(\left(\omega_{q2} - \omega_{q1} - \alpha_1 - \nu_r\right)t\right) \\ &+ \frac{W_r}{\sqrt{2}} \cos\left(\left(\omega_{q2} - \omega_{q1} + \alpha_2 + \nu_r\right)t\right) \\ &+ W_b \cos\left(\left(\omega_{q1} + \omega_{q2} - \nu_b\right)t\right) \\ &+ \frac{W_b}{2} \cos\left(\left(\omega_{q1} + \omega_{q2} + \alpha_1 + \alpha_2 + \nu_b\right)t\right), \end{aligned} \tag{23}$$

$$A_{QRj}(t) = \frac{\Omega_j}{\sqrt{2}} \cos\left(\left(\omega_{qj} + \omega_{rj} + \alpha_j\right)t\right). \tag{24}$$

Here $a_{qj(rj)}$ represents the annihilation operator for the j-th transmon (resonator); ω_{qj}, ω_{rj}, and α_j $(j = 1,2)$ are separately transmon's $|e\rangle \to |g\rangle$ transition frequency, transmon's anharmonicity, and resonator frequency. The QQ sideband modulation rates $\{\frac{W_r}{\sqrt{2}}, \frac{W_r}{\sqrt{2}}, W_b, \frac{W_b}{2}\}$ are scaled in the physical drives for equal interaction strength in the rotating frame. In all the discussion below, we require the interaction hierarchy $\alpha_j \gg W_{r,b} \sim \nu_{r,b} \gg \Omega_j$

Next, we move to the "*logical-static*" frame where all logical states have zero energy. The Hamiltonian $\tilde{H}_{\text{static}}$

becomes (we keep the lowest two levels for the resonators)

$$\tilde{H}_{\text{static}} = H_c + \tilde{H}_{QQ} - \sum_{j=1,2}\left(\frac{\alpha_j}{2}a_{rj}^\dagger a_{rj} + \tilde{H}_{QRj}\right), \quad (25)$$

$$H_c = -\frac{\alpha_1}{2}(P_{eg}+P_{ef}) - \frac{\alpha_2}{2}(P_{ge}+P_{fe}) \quad (26)$$

$$\begin{aligned}\tilde{H}_{QQ} =& \frac{W}{2}\left(|ee\rangle\langle gf| e^{2\pi i\nu_r t} + |ee\rangle\langle fg| e^{2\pi i\nu_r t}\right.\\ &\left. + |ee\rangle\langle gg| e^{2\pi i\nu_b t} + |ee\rangle\langle ff| e^{2\pi i\nu_b t}\right)\otimes I_4\\ &+ h.c., \end{aligned} \quad (27)$$

$$\tilde{H}_{QR1} = \frac{\Omega_1}{2}\left(|eg\rangle\langle fg| + |ef\rangle\langle ff|\right)\otimes|0\rangle\langle 1|\otimes I_2 + h.c., \quad (28)$$

$$\tilde{H}_{QR2} = \frac{\Omega_2}{2}\left(|ge\rangle\langle gf| + |fe\rangle\langle ff|\right)\otimes I_2\otimes|0\rangle\langle 1| + h.c.. \quad (29)$$

Here $P_{ab} = |ab\rangle\langle ab|\otimes I_4$. The transformation unitary $U_{\text{rot}} = U_5U_2U_1$ is a product of 3 unitaries:

$$U_1(t) = \exp\left[i\sum_{j=1,2}\left(\omega_{qj}+\frac{\alpha_j}{2}\right)a_{qj}^\dagger a_{qj}t\right], \quad (30)$$

$$U_2(t) = \exp\left[-i\frac{\alpha_1+\alpha_2}{2}P_{ee}t\right], \quad (31)$$

$$U_5(t) = \exp\left[i\sum_{j=1,2}(\omega_{rj}+\frac{\alpha_j}{2})a_{rj}^\dagger a_{rj}t\right]. \quad (32)$$

We define the other two unitaries U_3, U_4 for removing all time-dependency in the previous frame.

$$U_3(t) = \exp\left[i\nu_r(P_{gf}+P_{fg}+P_{ge}+P_{eg})t\right], \quad (33)$$
$$U_4(t) = \exp\left[i\nu_b(P_{gg}+P_{ff}+P_{ef}+P_{fe})t\right], \quad (34)$$

Choosing the rotating unitary $\tilde{U}_{\text{rot}} = U_5U_4U_3U_2U_1$ with the other two shown in Supplementary Eq. (33) and (34), all detuned QQ sidebands become time-independent. This leads to the Hamiltonian $\tilde{H}_{\text{rot}}$ in the fully rotated frame

$$\begin{aligned}\tilde{H}_{\text{rot}} =& -\frac{\alpha_1}{2}(P_{eg}+P_{ef}) - \frac{\alpha_2}{2}(P_{ge}+P_{fe})\\ &-\nu_r(P_{gf}+P_{fg}+P_{ge}+P_{eg})\\ &-\nu_b(P_{gg}+P_{ff}+P_{ef}+P_{fe})\\ &+\tilde{H}'_{QQ}\\ &-\sum_{j=1,2}\frac{\alpha_j}{2}a_{rj}^\dagger a_{rj} + \tilde{H}_{QRj}, \end{aligned} \quad (35)$$

$$\begin{aligned}\tilde{H}'_{QQ} =& \frac{W}{2}\left(|ee\rangle\langle gf| + |ee\rangle\langle fg|\right.\\ &\left.+|ee\rangle\langle gg| + |ee\rangle\langle ff| + h.c.\right)\otimes I_4. \end{aligned} \quad (36)$$

Here I_n is the $n\times n$ identity matrix. Rotating Wave Approximation (RWA) is applied in the last two transformations U_3 and U_4. In the final frame, $\{|L_0\rangle, |L_1\rangle\}$ have different energies $\{-\nu_r, -\nu_b\}$, and the superposition states become time-dependent.

Supplementary Fig. 4 shows the energy diagram at the fully rotated frame when treating QR interactions as perturbations. By diagonalizing Supplementary Eq. (35), energies for nine eigenstates with no photon in the resonators are explicitly labeled in the figure. This can be classified into three sets of states:

(a) The logical basis $\{|L_0\rangle, |L_1\rangle\}$
(b) The error states $\{|eg\rangle, |ef\rangle, |ge\rangle, |fe\rangle\}$
(c) The quasi-stable states $\{|S_\pm\rangle, |T\rangle\}$

The state energies for $\{|S_+\rangle, |T\rangle, |S_-\rangle\}$ are $\{x_1, x_2, x_3\}$, which are the roots ($x_1 > x_2 > x_3$) of the equation

$$\begin{aligned}&\frac{x^3}{8} + (\nu_r+\nu_b)\frac{x^2}{2} + (-W_r{}^2 - W_b{}^2 + 2\nu_r\nu_b)x\\ &-4W_r{}^2\nu_r - 4W_b{}^2\nu_b = 0. \end{aligned} \quad (37)$$

Star code requires no energy degeneracy between the logical states and any other states in the system. Energy degeneracy between $\{|L_0\rangle, |L_1\rangle, |T\rangle\}$ are avoided by choosing different ν_r and ν_b.

B. States Transition Rate

Logical lifetime is calculated analytically given the level diagram in Supplementary Fig. 4. Assuming only photon loss (dephasing is suppressed), there are two uncorrectable error channels: a second photon loss before the error correction and the population trapped in the quasi-stable state. All error correction is a two-step refilling process. For Q_1's photon loss error, the refilling rate from $|eg00\rangle$ to $|L_000\rangle$ and from $|ef00\rangle$ to $|L_100\rangle$ are both $\Gamma_{R1} = \frac{\Omega_1^2\kappa_1}{\Omega_1^2+\kappa_1^2}$. Due to the finite energy gap, the QR sideband also introduces a slow oscillation between $|eg00\rangle$ and $\{|S_-10\rangle, |T10\rangle, |S_+10\rangle\}$ (discussions for $|ef00\rangle$ is similar). Once the photon in the resonator decays, the population is trapped in $|S_-00\rangle$, $|T00\rangle$, $|S_+00\rangle$, causing the logical decoherence. The quasi-stable states' refilling rate $\{\Gamma_{S-}^{L0;R1}, \Gamma_T^{L0;R1}, \Gamma_{S+}^{L0;R1}\}$ are also two-step refilling processes:

$$\begin{cases}\Gamma_{S-}^{L0;R1} = \frac{\kappa_1\Omega_1^2(\langle S_-||fg\rangle)^2}{\kappa_1^2+\Omega_1^2(\langle S_-||fg\rangle)^2+4(x_3+\nu_r)^2},\\ \Gamma_T^{L0;R1} = \frac{\kappa_1\Omega_1^2(\langle T||fg\rangle)^2}{\kappa_1^2+\Omega_1^2(\langle T||fg\rangle)^2+4(x_2+\nu_r)^2},\\ \Gamma_{S+}^{L0;R1} = \frac{\kappa_1\Omega_1^2(\langle S_+||fg\rangle)^2}{\kappa_1^2+\Omega_1^2(\langle S_+||fg\rangle)^2+4(x_1+\nu_r)^2}.\end{cases} \quad (38)$$

The refilling rate superscript means when $|L_0\rangle$ is the initial state, using R_1 to correct photon loss in Q_1. The subscript shows the final state. The other three sets of refilling rates $\{\Gamma_{S-}^{L0;R2}, \Gamma_T^{L0;R2}, \Gamma_{S+}^{L0;R2}\}$, $\{\Gamma_{S-}^{L1;R1}, \Gamma_T^{L1;R1}, \Gamma_{S+}^{L1;R1}\}$, $\{\Gamma_{S-}^{L1;R2}, \Gamma_T^{L1;R2}, \Gamma_{S+}^{L1;R2}\}$ have a similar expression.

C. Code Performance

For simplicity, suppose both transmons have the same $|e\rangle \to |g\rangle$ decay rate γ, $\Gamma_{Rj} = \Gamma_R$, and $\Gamma^{Lk;Rj}_{S_-/T/S_+} = \Gamma^{Lk}_{S_-/T/S_+}, (j = 1, 2; k = 0, 1)$. The logical depolarization rate $\Gamma_Z = \Gamma_{L_0} + \Gamma_{L_1}$ is estimated classically through the Markov process of the state population.

$$\begin{cases} \frac{dP_{L0}(t)}{dt} = & -2\gamma P_L(t) + 2\Gamma_R P_{E0}(t) \\ \frac{dP_{E0}(t)}{dt} = & \gamma P_{L0}(t) \\ & -\left(\gamma + \Gamma_R + \Gamma^{L0}_{S-} + \Gamma^{L0}_{T} + \Gamma^{L0}_{S+}\right) P_{E0}(t) \, . \\ P_{L0}(0) = & 1 \\ P_{E0}(0) = & 0 \end{cases} \tag{39}$$

Here $P_{L0}(t)$ and $P_{E0}(t)$ are the state population at $|L_0 00\rangle$ and the error state manifold $\{|eg00\rangle, |ge00\rangle\}$ at time t. As a modest lower-bound estimation, we do not consider the population after being trapped in the quasi-stable states. The solution of P_{L0} is a sum of two exponential decays. The fast one represents the transition period behavior, and the slow decay constant is the actual decay rate Γ_{L0}

$$\Gamma_{L0} \sim \frac{2\gamma(\gamma + \Gamma^{L0}_{S_-} + \Gamma^{L0}_{T} + \Gamma^{L0}_{S_+})}{3\gamma + \Gamma_R + \Gamma^{L0}_{S_-} + \Gamma^{L0}_{T} + \Gamma^{L0}_{S_+}}. \tag{40}$$

Similarly the decay rate for $|L_1\rangle$ is

$$\Gamma_{L1} \sim \frac{2\gamma(3\gamma + \Gamma^{L1}_{S_-} + \Gamma^{L1}_{T} + \Gamma^{L1}_{S_+})}{5\gamma + \Gamma_R + \Gamma^{L1}_{S_-} + \Gamma^{L1}_{T} + \Gamma^{L1}_{S_+}}. \tag{41}$$

The logical state lifetime for both $|L_0\rangle$ and $|L_1\rangle$ is $1/(\Gamma_{L_0} + \Gamma_{L_1})$. Given $\Gamma_R \gg \gamma \gg \Gamma^{L0/L1}_{S_-/T/S_+}$, the logical coherence approximately becomes $\Gamma_R/(8\gamma^2)$. Therefore the Star code shows a quadratic improvement over the single photon loss rate γ.

$$\langle L_x| a_{qj} a_{qj} |L_x\rangle = 1/\sqrt{2} \tag{42}$$

$$\langle L_y| a_{qj} a_{qj} |L_y\rangle = 0 \tag{43}$$

For the superposition states $|L_x\rangle = (|L_0\rangle + |L_1\rangle)/\sqrt{2}$, there are extra logical coherence protections coming from the code structure. When the double-photon-loss error happens on the same transmon (Supplementary Eq. (42)), the final state recovers part of the $|L_x\rangle$ (50% in fidelity). Given $\Gamma_R \gg \gamma \gg \Gamma^{L0/L1}_{S_-/T/S_+}$, lifetime ratio between $|L_x\rangle$ and $|L_{0/1}\rangle$ is 4/3.

However, $|L_y\rangle = (|L_0\rangle + 1j\,|L_1\rangle)/\sqrt{2}$ does not enjoy the extra protection (Supplementary Eq. (43)). Therefore $\{|L_0\rangle, |L_1\rangle, |L_y\rangle\}$ have the same lifetime while $|L_x\rangle$ is longer in the ideal case. Numerical simulations for $\{|L_0\rangle, |L_1\rangle, |L_y\rangle, |L_x\rangle\}$ are shown in the Supplement Note D.

Transition label	Description	Rate (kHz)
Γ_{R1}	Q_1 decay error correction rate	186.2
Γ_{R2}	Q_2 decay error correction rate	190.9
$\Gamma^{L0;R1}_{S-}$	$\|L_0\rangle \to \|S_-\rangle$ rate (Q_1 decay)	8.0
$\Gamma^{L0;R1}_{T}$	$\|L_0\rangle \to \|T\rangle$ rate (Q_1 decay)	3.7
$\Gamma^{L0;R1}_{S+}$	$\|L_0\rangle \to \|S_+\rangle$ rate (Q_1 decay)	0.1
$\Gamma^{L0;R2}_{S-}$	$\|L_0\rangle \to \|S_-\rangle$ rate (Q_2 decay)	7.4
$\Gamma^{L0;R2}_{T}$	$\|L_0\rangle \to \|T\rangle$ rate (Q_2 decay)	3.4
$\Gamma^{L0;R2}_{S+}$	$\|L_0\rangle \to \|S_+\rangle$ rate (Q_2 decay)	1.0
$\Gamma^{L1;R1}_{S-}$	$\|L_1\rangle \to \|S_-\rangle$ rate (Q_1 decay)	0.1
$\Gamma^{L1;R1}_{T}$	$\|L_1\rangle \to \|T\rangle$ rate (Q_1 decay)	6.0
$\Gamma^{L1;R1}_{S+}$	$\|L_1\rangle \to \|S_+\rangle$ rate (Q_1 decay)	10.7
$\Gamma^{L1;R2}_{S-}$	$\|L_1\rangle \to \|S_-\rangle$ rate (Q_2 decay)	0.1
$\Gamma^{L1;R2}_{T}$	$\|L_1\rangle \to \|T\rangle$ rate (Q_2 decay)	5.1
$\Gamma^{L1;R2}_{S+}$	$\|L_1\rangle \to \|S_+\rangle$ rate (Q_2 decay)	10.0

Supplementary Table 4. Calculated refilling process rate in our experiment. Logical refilling rate $\{\Gamma_{R1}, \Gamma_{R2}\}$ dominants over other logical error rate.

The error correction performance benefits from larger Γ_{Rj} and smaller quasi-stable states' refiling rates. Γ_{Rj} are increased with Ω_j, which is bounded by $W_{r/b}$ for perturbation validity. Quasi-stable states' refiling rates are suppressed with larger $W_{r/b}$ and $\nu_{r,b}$. From Supplementary Eq. (38), quasi-stable states' refilling rates are quadratically reduced with a larger energy gap $|x_j + \nu_{r,b}|$. Increasing $W_{r,b}$ and $\nu_{r,b}$ generally helps increase the energy gaps $|x_j + \nu_{r,b}|$. And as a special case, when $\nu_r = -\nu_b = \nu$, $W_r = W_b = W$, $\{x_j\}$ is $\{-\sqrt{W^2+\nu^2}, 0, \sqrt{W^2+\nu^2}\}$. Clearly, the energy gap is increased with larger W and ν.

With our experiment parameters

$$\{W_r, W_b, \Omega_1, \Omega_2, \kappa_1, \kappa_2, \nu_r, \nu_b\} \\ = \{1.45, 1.25, 0.39, 0.39, 0.53, 0.48, 0.8, -0.9\} \text{ MHz},$$

The quasi-stable states $\{|S_\pm, |T\rangle\rangle\}$ have the following overlap with the basis $\{|fg\rangle, |gg\rangle\}$:

$$\{(\langle S_-| \, |fg\rangle)^2, (\langle T| \, |fg\rangle)^2, (\langle S_+| \, |fg\rangle)^2\} \\ = \{0.294, 0.174, 0.032\}, \tag{44}$$

$$\{(\langle S_-| \, |gg\rangle)^2, (\langle T| \, |gg\rangle)^2, (\langle S_+| \, |gg\rangle)^2\} \\ = \{0.023, 0.185, 0.293\} \tag{45}$$

All refilling process rates are shown in Supplementary Table 4.

D. Effects From Stray ZZ Couplings

In our experiment, the ZZ couplings in each transmon-resonator pair and between two transmons play differ-

ent roles in the Star code. As the resonator photon energy remains the same for all logical states, the resonator photon carries no logical information in the presence of transmon-resonator ZZ coupling and is irrelevant to the logical coherence.

However, ZZ coupling between two transmons reduces coherence for any logical superposition states. In the presence of transmons ZZ coupling. photon lost from one of the transmons carries different energies conditioned on the logical state. As a result, $|L_1\rangle$ have a different phase evolution compared to $|L_0\rangle$ after error correction. The phase is stochastic and dephases any superposition state. In the experiment, the ZZ coupling error dominates over all other error channels and results in a very similar but slightly less lifetime for $|L_y\rangle$ than $|L_x\rangle$. Since $|L_0\rangle$ and $|L_1\rangle$ are not affected by ZZ, the lifetime is higher than both $|L_x\rangle$ and $|L_y\rangle$. Detailed experimental error channel is shown in Supplementary Note 10.

Our inductive coupler suppresses the ZZ between transmons by choosing an optimal DC flux. ZZ cancellation is critical for improving coherence for the superposition of logical states and requires further experimental improvements. Further ZZ cancellation can be achieved by adding additional flux drives to the coupler. Details are discussed in Supplementary Note 4.

SUPPLEMENTARY NOTE 4: TRANSMON ZZ INDUCED DEPHASING

Realizing AQEC requires error transparency to single photon loss error. This makes ZZ coupling an extra logical dephasing channel as it does not commute with $\tilde{H}_{\text{star}}$. In a two qutrit system, there are in total 7 different ZZ frequency shifts coming from 4 cross-Kerr coupling strengths $J_{11}, J_{21}, J_{12}, J_{22}$. However, not all ZZ couplings are detrimental to the Star code. The error transparency requires no phase accumulation between logical states during the error correction process. This is equivalent to having the same energy for the photon lost from one transmon, independent of the state of the partner transmon,

$$\begin{aligned} E_{ff} - E_{ef} &= E_{fg} - E_{eg}, \\ E_{ff} - E_{fe} &= E_{gf} - E_{ge}. \end{aligned} \tag{46}$$

Here E_{jk} refers to the energy of the state $|jk\rangle$. Supplementary Eq. (46) are equivalent to $ZZ_{ff2} = ZZ_{ff1} = 0$ (see Supplementary Table 2). When this is not the case, a random phase difference will accumulate between logical states after the error correction, introducing dephasing to the logical superposition states. The other ZZs are naturally error transparent in the logical manifold since $|ee\rangle$ is dark and will not affect the logical states' coherence. To suppress the ZZ-induced logical dephasing, we can increase the QR sideband rate Ω_j, shortening the $|e\rangle$ population time in both qutrits and reducing the accumulated random logical phase. To eliminate this dephasing channel, we need a coupler that has zero ZZ_{ff1}

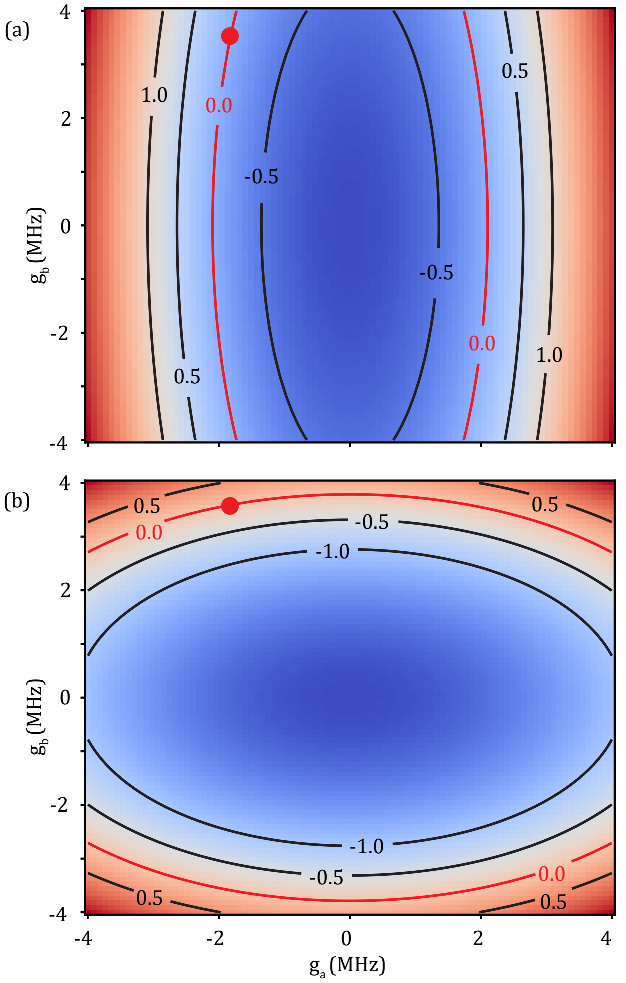

Supplementary Figure 5. ZZ cancellation. Total ZZ_{ff1} (a) and ZZ_{ff2} (b) (in MHz) are shown in the contour while sweeping amplitudes of two extra detuned external drives ($|ef\rangle \leftrightarrow |gh\rangle$ and $|fe\rangle \leftrightarrow |hg\rangle$). Individual ZZ cancellation contour line (with value 0) is marked. One of the four simultaneous cancellation points is highlighted in red circle.

and ZZ_{ff2} when all external sidebands are turned on. This can be potentially realized in our current coupler with dispersive shift engineering. We consider a two-transmon system with static interaction that produces a set of dispersive shifts for cancellation. The base Hamiltonian H_{base} is

$$\begin{aligned} H_{\text{base}} = &\sum_n \left(\epsilon_{1,n} |n_1\rangle\langle n_1| + \epsilon_{2,n} |n_2\rangle\langle n_2|\right) \\ &+ \sum_{nm} \Delta_{nm} |n_1 m_2\rangle\langle n_2 m_1|, \end{aligned} \tag{47}$$

where $\epsilon_{1/2,n}$ are energies for single transmon levels, Δ_{nm} is the static energy shift to the state when transmon 1 has n photons and transmon 2 has m photons. For the ground state $\epsilon_{1/2,0}$ and Δ_{00} are set to 0. We add to H_{base}

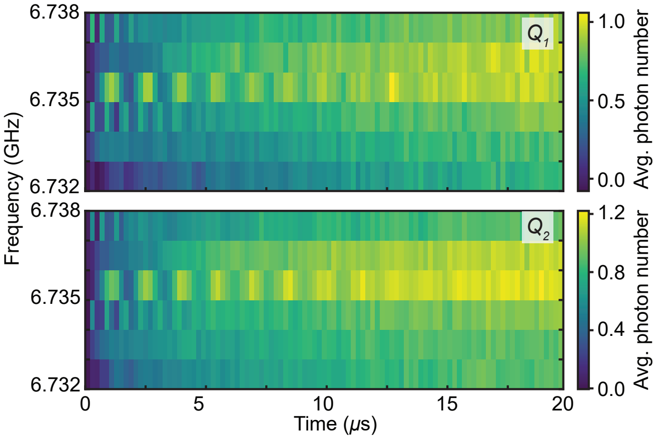

Supplementary Figure 6. Readout saturation feature for fast (rate 9 MHz) QQ blue sideband $|gg\rangle \leftrightarrow |ee\rangle$ in the long time scale. Top and bottom panel show readout signals from the first and second resonators.

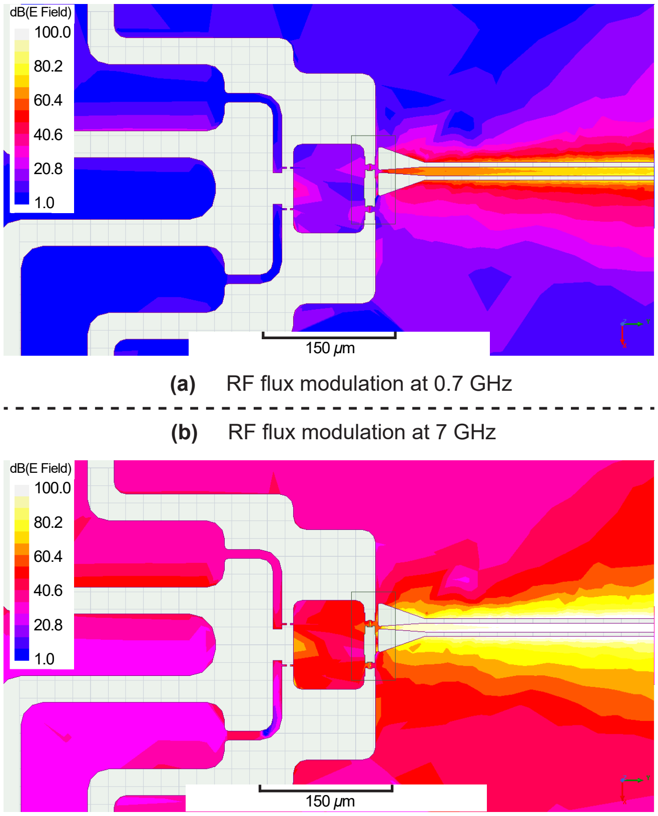

Supplementary Figure 7. Distribution of electrical field's amplitude (in log scale) when RF flux drive is modulated at (a) 700 MHz and (b) 7 GHz obtained from HFSS simulation.

a QQ red sideband through the coupler,

$$H_D = 2g \sin(2\pi\nu t)\left(a_{q1}^\dagger a_{q2} + a_{q1} a_{q2}^\dagger\right). \tag{48}$$

We assume the frequency detuning ν is far off-resonant so that $\nu \gg g$, and this will introduce an energy shift D_{jk} to all levels:

$$D_{jk}^{(R)} = \frac{g^2 j(k+1)}{E_{j,k} - E_{j-1,k+1} - \nu} + \frac{g^2 j(k+1)}{E_{j,k} - E_{j-1,k+1} + \nu} + \frac{g^2 (j+1)k}{E_{j,k} - E_{j+1,k-1} - \nu} + \frac{g^2 (j+1)k}{E_{j,k} - E_{j+1,k-1} + \nu}. \tag{49}$$

For a detuned QQ blue sideband drive, one can find a similar expression for the energy shift,

$$D_{jk}^{(B)} = \frac{g^2 (j+1)(k+1)}{E_{j,k} - E_{j+1,k+1} - \nu} + \frac{g^2 (j+1)(k+1)}{E_{j,k} - E_{j+1,k+1} + \nu} + \frac{g^2 jk}{E_{j,k} - E_{j-1,k-1} - \nu} + \frac{g^2 jk}{E_{j,k} - E_{j-1,k-1} + \nu}. \tag{50}$$

When multiple external QQ sidebands are applied, the total dispersive shift to each energy level is given by $D_{jk} = \sum D_{jk}^{(R)} + \sum D_{jk}^{(B)}$, where the sum is over all external QQ sidebands. Specifically for the Star code scheme, we can modulate two extra QQ sidebands near $|ef\rangle \leftrightarrow |gh\rangle$ and $|fe\rangle \leftrightarrow |hg\rangle$. This can introduce either positive or negative D_{12} and D_{21} to the system, depending on the choices of frequency detuning. The required g for complete ZZ cancellation can be less than 5 MHz. Therefore, it is theoretically possible to cancel ZZ_{ff1} and ZZ_{ff2} simultaneously under such two extra drives.

We choose the experiment sideband parameters $\{W_r, W_b, \nu_r, \nu_b\} = \{1.45, 1.25, -0.8, 0.9\}$ MHz and current total ZZ rate $\{ZZ_{ff1}, ZZ_{ff2}\} = \{-0.6, -2.2\}$ MHz. We add two external drives: $|ef\rangle \leftrightarrow |gh\rangle$ and $|fe\rangle \leftrightarrow |hg\rangle$ with frequency detunings $\{-2, 2\}$ MHz and sweep the drive strengths $\{g_a, g_b\}$. Here $\{g_a, g_b\}$ is the coefficient g for H_D (Supplementary Eq. (48)). We calculated the total ZZ_{ff1} and ZZ_{ff2} based on Supplementary Eq. (49) and Supplementary Eq. (50) and plot the 2D sweep results in the following Supplementary Fig. 5. The simultaneous ZZ cancellation points are highlighted in the figure. Therefore it is always possible to fully cancel ZZ_{ff1} and ZZ_{ff2} with external drives. We did not turn on the cancellation sidebands in the experiment, because our readout suffers from frequency shift under strong flux modulation amplitude as discussed in Supplementary Note 5.

SUPPLEMENTARY NOTE 5: READOUT SATURATION AND FLUX LINE OPTIMIZATION

In our device, we use the on-chip flux line to generate various two-qubit interactions through parametric modulation. A typical parametric coupler design includes two qubits capacitively or inductively coupled through a tunable coupler. Modulation of the coupler frequency and the qubit-coupler coupling strength contribute to the two-qubit interaction strength. For a capacitively coupled system [3, 5–7], coupler frequency modulation con-

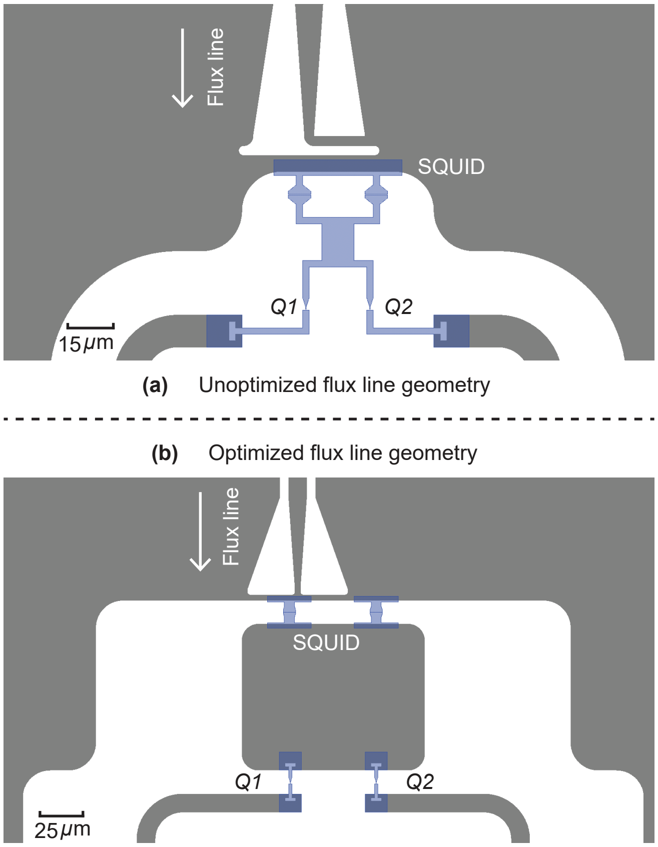

Supplementary Figure 8. SQUID design. (a) Old design without optimization. (b) Current design by maximizing the mutual inductance between the flux line and SQUID. The Ta and Al areas are colored as grey and blue separately, and the bare sapphire is colored white.

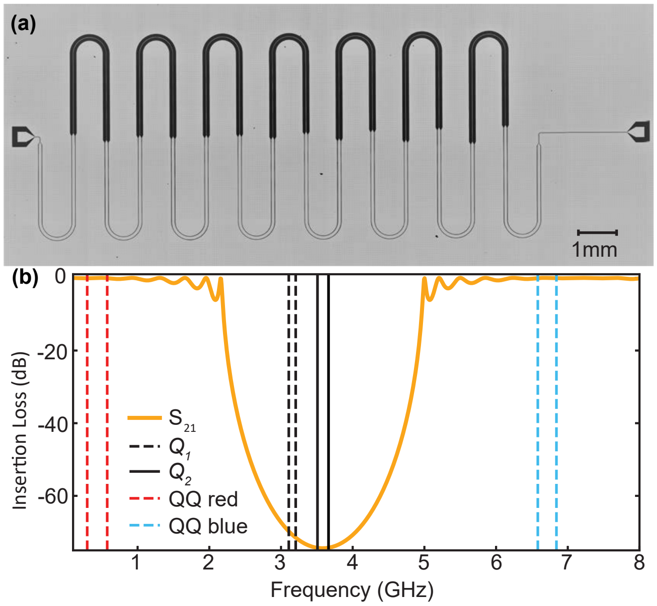

Supplementary Figure 9. The stepped impedance Purcell filter (SIPF). (a) Optical image of our SIPF chip made using Ta on sapphire. (b) Calculated SIPF insertion loss. Transmon transitions, QQ red and blue sideband frequencies are marked in the plot.

tributes dominantly to the QQ sideband rate, and time-dependent Schrieffer-Wolff transformation (SWT) proves that [3] the ratio of interaction strengths between bswap and iswap is $\frac{\omega_1-\omega_2}{\omega_1+\omega_2}$ (ω_j is Q_j's frequency). Therefore, a capacitive coupler provides a slower bswap than the iswap. In contrast, an inductively coupled system [2, 8] modulates the coupling strength between the qubit and coupler more effectively, and both iswap and bswap will have the same zeroth-order terms in the SWT expansion, thus theoretically sharing the same rate under same modulation amplitude. Previous experiments achieved fast iswap interactions, but a similar bswap rate has not been demonstrated in either type of parametric coupler yet. We experimentally realize a comparable maximum rates of 9 MHz bswap and 21 MHz iswap (shown in Supplementary Fig. 3).

In the experiment, we notice that turning a strong bswap on will shift both resonators' frequencies after a long time, resulting in the 'saturation' feature (Supplementary Fig. 6). Such a readout frequency shift is both sideband strength and duration dependent, and the shift persists for a noticeable period after all sidebands are turned off. Distinguishing transmons' states through readout becomes difficult when this happens. A readout is possible when the shift is reversed after waiting for a sufficiently long period but degrades readout fidelity due to transmons' relaxation. While case-dependent dynamic demarcation can distinguish states, this method becomes complex and inaccurate. In our experiment, we decided to lower the RF modulation amplitude and minimize the saturation region by optimizing the flux line geometry.

One source for the readout saturation at the bswap drive frequency is the flux line's stray charge coupling to the SQUID [8]. The on-chip flux line can be considered an antenna. The coupler is located in the near-field region, and the electrical field amplitude is proportional to the flux modulation frequency. Since bswap's drive frequencies are normally a magnitude higher than that of the iswap operations, a much stronger stray-charge drive is observed when the bswap drive is on.

We verify this fact using ANSYS HFSS simulation (see Supplementary Fig. 7), where the electrical field amplitude is observed to increase over an order of magnitude when the modulation frequency is increased by a factor of 10. The stray charge drive limits the maximum power we can use for the flux modulation, and we focus on geometrical optimization to improve the flux-to-charge drive ratio. In order to do so, we maximize the mutual inductance between the SQUID and the flux line by increasing the SQUID loop size and bringing the flux line closer to the loop. The loop size in our experiment is limited by the SQUID's hysteresis [9] set by the ratio $\frac{L_{\text{loop}}}{L_{jc}}$, where L_{jc} is the inductance of each junction in

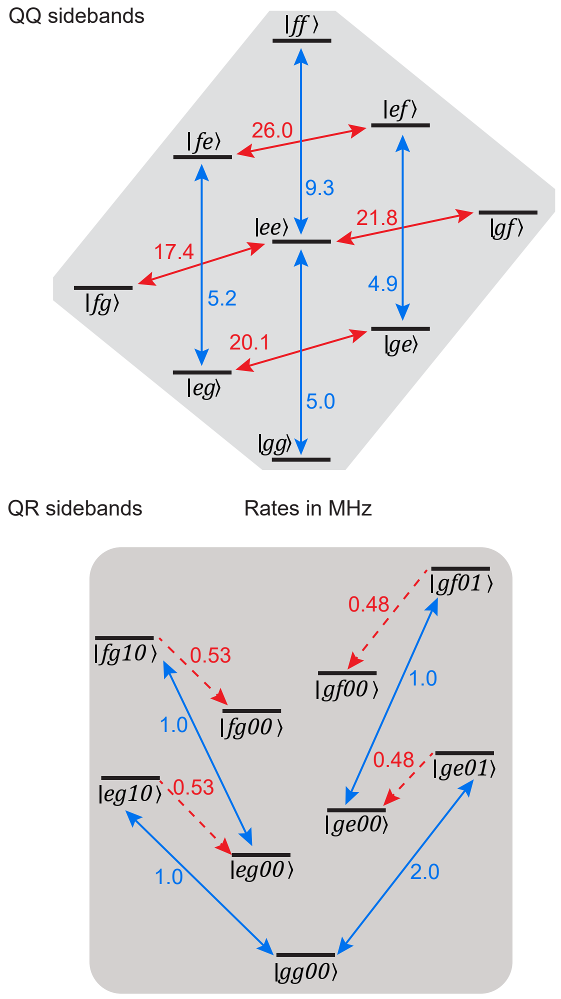

Supplementary Figure 10. QQ (top panel) and QR (bottom panel) sidebands with rates achieved in the experiment without readout saturation.

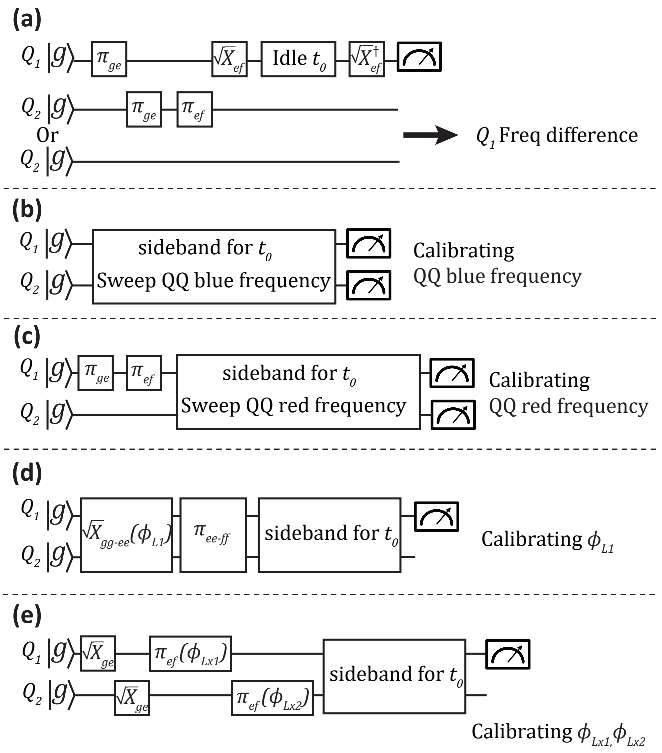

Supplementary Figure 11. Gate circuits for the Star code calibration. (a) Pulse sequence for calibration of ZZ_{ff1}. Ramsey-like protocol is used for the partner transmon being in $|g\rangle$ or $|f\rangle$. The other ZZs are calibrated similarly. (b) QQ blue and (c) QQ red sideband frequency calibration sequence. QQ sideband pair frequencies are iteratively swept and updated based on the time-domain pattern. (d) $|L_1\rangle$ preparation phase calibration sequence. The phase point that has minimum $|e\rangle$ population in the sweep is chosen as the calibrated ϕ_{L1}. The preparation phase for $|L_0\rangle$ is calibrated similarly. (e) $|L_x\rangle$ preparation phase calibration protocol. All $\sqrt{X}$ operators represent $\pi/2$ rotations.

the SQUID (assumed identical) and L_{loop} is the SQUID loop inductance. When $\frac{L_{\text{loop}}}{L_{jc}} > 1$, transmon frequencies become hysteric as a function of Φ_{DC}, and the region grows with the ratio. Dissipation appears when modulating within the hysteric region and should be avoided in our experiment. This property sets an upper bound for SQUID loop length.

We use HFSS simulation to calculate flux threaded by the SQUID loop and vary the geometry to maximize. Assuming the electrical field is geometry insensitive around the SQUID, maximizing SQUID flux increases the ratio between the mutual inductive coupling and stray capacitive coupling strength of the flux line. The original and optimized designs are shown in Supplementary Fig. 8. The simulation suggests a factor of 3.5 improvement in the ratio.

Being strongly coupled to the SQUID, the flux line is also a channel for transmons' relaxation. In order to improve Purcell protection, we design a Stepped-Impedance band-stop Purcell Filter (SIPF) as shown in Supplementary Fig. 9, which strongly blocks transmon frequencies while allowing the QQ red and blue sideband drives to pass (see Supplementary Fig. 22 for the full measurement setup).

SUPPLEMENTARY NOTE 6: QQ AND QR SIDEBAND RATES

The ability to sustain fast QQ sidebands is crucial to the realization of the Star code, and we optimize the geometry of the device as discussed in the previous section. In Supplementary Fig. 10(a), we show all two-photon QQ sidebands achieved in the experiment. Supplementary Fig. 10(b) shows all experimentally achieved QR sidebands and resonator decay rates. The strong and rich two-photon processes with this design also show the potential of realizing high-fidelity two-qutrit gates [10].

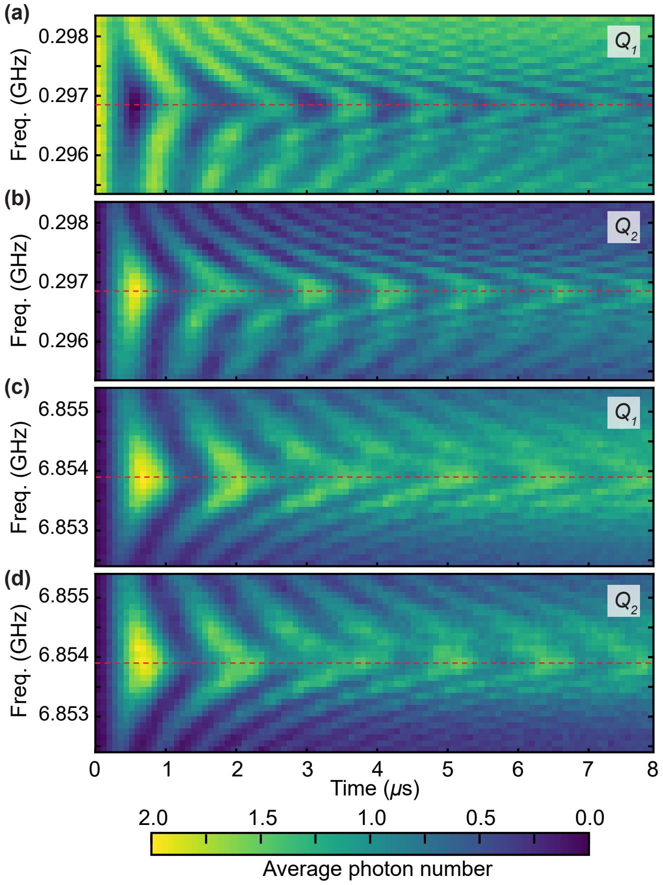

Supplementary Figure 12. Simultaneous QQ sideband calibration. All six tones used in the Star code are simultaneously turned on during the frequency-time sweep. The red (top two panels) and blue (bottom two panels) QQ sideband pair frequencies are swept individually for calibration in the presence of the other pair. Red dash lines in the plots represent the pairs' center frequency choices. (a) and (b) are Q_1 and Q_2's average photon number when sweeping the red pair with the initial state $|fg\rangle$. (c) and (d) are Q_1 and Q_2's average photon number when sweeping the blue pair with the initial state $|gg\rangle$.

We list all two-photon sideband theoretical transition frequencies within the first four levels (g,e,f,h) of both transmons in Supplementary Table 5. The calculation is based on bare transmon frequency levels and ZZ coupling between transmons. The two minimum frequency collisions are between $|fg\rangle \leftrightarrow |ee\rangle$ and $|he\rangle \leftrightarrow |ff\rangle$ (43.7 MHz), and between $|ee\rangle \leftrightarrow |ff\rangle$ and $|gf\rangle \leftrightarrow |eh\rangle$ (43.2 MHz). When $W \sim 1.5$ MHz, the $|h\rangle$ population rate is less than 0.15% of logical state refilling rate Γ_R, or at most 0.27 kHz. Therefore, we assume that the $|h\rangle$ state leakage is unimportant in our experiment.

Transition	Freq (MHz)	Transition	Freq (MHz)
$\|ge\rangle \leftrightarrow \|eg\rangle$	457.6	$\|gg\rangle \leftrightarrow \|ee\rangle$	6867.0
$\|fg\rangle \leftrightarrow \|ee\rangle$	573.6	$\|eg\rangle \leftrightarrow \|fe\rangle$	6750.2
$\|ee\rangle \leftrightarrow \|gf\rangle$	298.4	$\|ge\rangle \leftrightarrow \|ef\rangle$	6706.9
$\|hg\rangle \leftrightarrow \|fe\rangle$	689.6	$\|fg\rangle \leftrightarrow \|he\rangle$	6633.4
$\|fe\rangle \leftrightarrow \|ef\rangle$	414.3	$\|ee\rangle \leftrightarrow \|ff\rangle$	6589.7
$\|ef\rangle \leftrightarrow \|gh\rangle$	139.3	$\|gf\rangle \leftrightarrow \|eh\rangle$	6546.5
$\|he\rangle \leftrightarrow \|ff\rangle$	529.9	$\|fe\rangle \leftrightarrow \|hf\rangle$	6472.2
$\|ff\rangle \leftrightarrow \|eh\rangle$	255.2	$\|ef\rangle \leftrightarrow \|fh\rangle$	6428.7
$\|hf\rangle \leftrightarrow \|fh\rangle$	370.8	$\|ff\rangle \leftrightarrow \|hh\rangle$	6310.6

Supplementary Table 5. All possible two-photon sideband theoretical transition frequencies between two transmons. Frequencies are based on static transmons levels at the same DC flux bias point in the experiment.

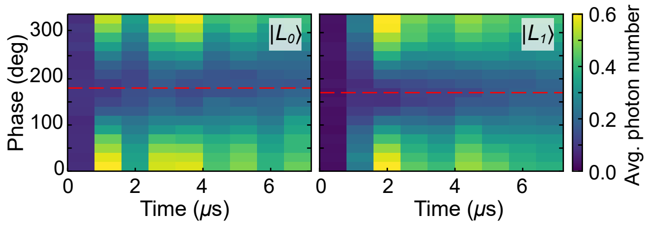

Supplementary Figure 13. Preparation phase calibration of $|L_0\rangle$ (left) and $|L_1\rangle$ (right) in the error correction experiment. The population of $|e\rangle$ on Q_1 are measured after turning on all sidebands for up to 8 µs, and the red dash line marks the calibrated phase position. We choose Q1 due to higher readout fidelity.

SUPPLEMENTARY NOTE 7: FULL STAR CODE CALIBRATION PROCESS

To implement the Star code, we need to calibrate the QQ and QR sideband frequencies when all sidebands are simultaneously on. The presence of external sidebands will change both transmons' frequencies through AC-stark shift and rectifying effect (RF modulation under a nonlinear frequency-flux response). In the experiment, we systematically perform the calibration, shown in Supplementary Fig. 11.

In Supplementary Fig. 11a, the static ZZ dispersive shift is characterized by measuring the Ramsey fringe frequency difference depending on the other qubit's state. In steps Supplementary Fig. 11b and Supplementary Fig. 11c, we first turn on all 6 QQ and QR drives at their bare frequencies. All QQ sideband rates are set to W when independently turned on. Two QQ red sidebands and two QQ blue sidebands are pair-swept separately as the 'red/blue pair'. The pair width and center are the sidebands' frequency difference and average. In each iteration step, we update sequentially the red and blue pair centers, and the QR sideband frequencies. For each pair, we sweep the center frequency as a function of time with all six sidebands on. We use $|gg\rangle$ (blue pair) and $|fg\rangle$ (red pair) as the initial states. Reading out the average photon number in both transmons, the 2D sweep plots

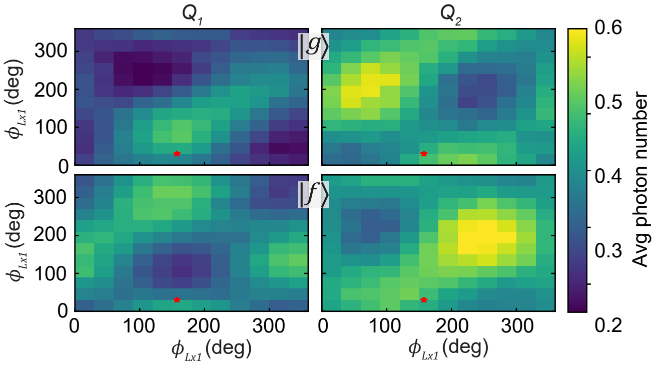

Supplementary Figure 14. Phase calibration for $|L_x\rangle$ preparation. Population of $|g\rangle$ (top) and $|f\rangle$ (bottom) on Q_1 (left) and Q_2 (right) are measured after 8 µs, and the red star marks the calibrated phase position.

show a fringed chevron pattern (shown in Supplementary Fig. 12). The pattern's center line is the new pair center. The fringe rate represents the actual sideband detunings $\nu_{b/r}$ and rate $W_{b/r}$, and the detunings can be updated by changing pair width at this stage. After extracting both pairs' new centers, the QR sidebands are calibrated with $|eg\rangle$ and $|ge\rangle$ as the initial state when all drives are on. Populating $|f\rangle$ with the $|e0\rangle \leftrightarrow |f1\rangle$ process is most efficient when QR sidebands are on resonance. Because of none zero ZZ_{ff1} and ZZ_{ff2}, the QR sidebands cannot be exactly on resonance for both $|L_i\rangle$. In the experiment, we calibrate QR sidebands to be on resonance for the $|L_0\rangle$. For $|L_1\rangle$, the error correction process will be slower but not dephase the state after correction. After a few iterations, we get decent frequency calibrations of all six sidebands.

Logical state preparation includes both charge and flux drives with appropriate relative phases. Supplementary Fig. 11d is to calibrate $|L_0\rangle$ and $|L_1\rangle$'s preparation phase. For the logical state $|L_0\rangle$, we first apply two π_{ge} pulses sequentially on Q_1 and Q_2 to prepare $|ee\rangle$ through charge lines. Afterwards a $(\pi/2)_{|ee\rangle\leftrightarrow|gf\rangle}$ pulse with a phase offset ϕ_{L0} and a $\pi_{|ee\rangle\leftrightarrow|fg\rangle}$ pulse are applied through the flux line. To prepare $|L_1\rangle$, a $(\pi/2)_{|gg\rangle\leftrightarrow|ee\rangle}$ pulse with some phase ϕ_{L1} followed by a $\pi_{|ee\rangle\leftrightarrow|ff\rangle}$ are applied through the flux line. These steps generally prepare $(|gf\rangle - e^{i\phi_{L0}}|fg\rangle)/\sqrt{2}$ and $(|gg\rangle - e^{i\phi_{L1}}|ff\rangle)/\sqrt{2}$. For non-zero $\{\phi_{L0}, \phi_{L1}\}$, $|ee\rangle$ is populated under the action of $\tilde{H}_{\text{static}}$, and we use this feature to find the correct preparation phases. We sweep the phase $\phi_{L0(1)}$ in the presence of all six tones and observe $|e\rangle$ population on both qutrits. The correct preparation phases are determined by values that minimize $|e\rangle$ population of both transmons during the first 8 µs of error correction, as presented in Supplementary Fig. 13.

Preparation of $|L_x\rangle = (|L_0\rangle + |L_1\rangle)/\sqrt{2}$ does not require any sideband pulses as it is a product state $(|g\rangle - |f\rangle)(|g\rangle + |f\rangle)/2$. We apply a $(\pi/2)_{ge}$ pulse with a specific phase, followed by a π_{ef} pulse on both transmons. These pulses prepare the state $\left(|g\rangle + e^{i\phi_{Lx1}}|f\rangle\right)\left(|g\rangle + e^{i\phi_{Lx2}}|f\rangle\right)/2$, leaving two preparation phases ϕ_{Lx1} and ϕ_{Lx2} left for calibration. The correct phase combination can be calibrated on the 2D ϕ_{Lx1}-ϕ_{Lx2} phase sweep plot. Correct preparation phases will keep equal populations of $|g\rangle$ and $|f\rangle$ for both transmons at any time after turning on all sidebands. In Supplementary Fig. 11e, both transmons' $|g\rangle$ and $|f\rangle$ populations are measured 8 µs after turning sidebands on. Supplementary Fig. 14 shows the 2D phase sweep plot. This yields four phase coordinates $\{\phi_{Lx1}, \phi_{Lx2}\} = \{0, 180°\} \otimes \{0, 180°\}$, and two of the four correspond to $(|L_0\rangle \pm |L_1\rangle)/\sqrt{2}$. We distinguish the logical $|L_x\rangle$ by taking two-qutrit state tomography measurements after turning on the sidebands for 9 µs and choose the error-corrected case. The calibration process for the 4 QQ echo case is the same, except the QR sidebands are off.

In the experiment, we pick the largest QQ sideband rate W that does not cause a significant heating effect and choose the other parameters $\{\nu_{r/b}, \Omega_{1/2}\}$ accordingly. As discussed in Section. , larger W means better AQEC performance. Given the maximum W fixed, the optimal QQ red and blue sideband detunings $\{\nu_r, \nu_b\}$ appear around $\nu_r = -\nu_b = \pm W/2$ [11]. In the experiment, we select the optimal sideband detunings before calibration, then extract the actual detunings (changed by AC stark shift) after the calibration $\{\nu_r, \nu_b\} = \{0.8, -0.9\}$ MHz. The AQEC performance is not sensitive to the detuning choice around the optimal point in the simulation. We choose the QR sideband rate Ω that has around the best AQEC performance in simulation given fixed W.

SUPPLEMENTARY NOTE 8: TWO QUTRIT TOMOGRAPHY

Following the basis choice in Ref. 12, we apply 81 post rotations S_j from the tomography rotation set $S \otimes S$: $S = \{I, R_{ge}\left(0, \frac{\pi}{2}\right), R_{ge}\left(\frac{\pi}{2}, \frac{\pi}{2}\right), R_{ge}\left(0, \pi\right), R_{ef}\left(0, \frac{\pi}{2}\right),$ $R_{ef}\left(\frac{\pi}{2}, \frac{\pi}{2}\right), R_{ef}\left(0, \frac{\pi}{2}\right) R_{ge}\left(0, \pi\right), R_{ef}\left(\frac{\pi}{2}, \frac{\pi}{2}\right) R_{ge}\left(0, \pi\right),$ $R_{ef}\left(0, \pi\right) R_{ge}\left(0, \pi\right)\}$. Here I is the identity gate, and the rotations R_{ge} and R_{ef} are defined as follows

$$R_{ge}(\phi, \theta) = \begin{bmatrix} \cos\frac{\theta}{2} & -e^{-i\phi}\sin\frac{\theta}{2} & 0 \\ e^{i\phi}\sin\frac{\theta}{2} & \cos\frac{\theta}{2} & 0 \\ 0 & 0 & 1 \end{bmatrix}, \quad (51)$$

$$R_{ef}(\phi, \theta) = \begin{bmatrix} 1 & 0 & 0 \\ 0 & \cos\frac{\theta}{2} & -e^{-i\phi}\sin\frac{\theta}{2} \\ 0 & e^{i\phi}\sin\frac{\theta}{2} & \cos\frac{\theta}{2} \end{bmatrix}. \quad (52)$$

Simultaneous single-shot readouts are collected after each of the 81 rotations. Supplementary Fig. 15 shows the single shot confusion matrix of our readout. To compensate for the measurement error, we applied the inverse of the confusion matrix to the readout result. Maximum-Likelihood-Estimation (MLE) is used to reconstruct the physical density matrix ρ_m that minimizes the cost func-

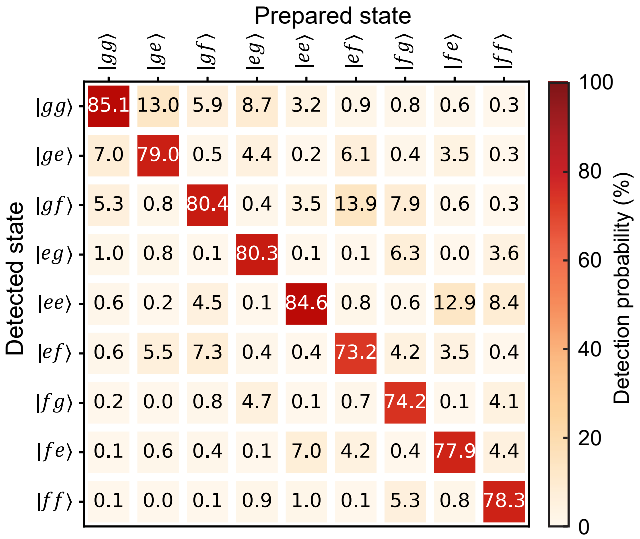

Supplementary Figure 15. Single shot confusion matrix. Two-qutrit basis states are prepared and measured 5000 times.

tion f_c,

$$f_c(\vec{p},\vec{q}) = \sum_{j=1}^{81} \sum_{a,b=g,e,f} \left(\frac{p_{j,|ab\rangle} - q_{j,|ab\rangle}}{q_{j,|ab\rangle}} \right)^2 ,$$
$$p_{j,|ab\rangle} = \langle ab| \, S_j \cdot \rho_m \, |ab\rangle ,$$
$$q_{j,|ab\rangle} = \langle ab| \, S_j \cdot \rho_{exp} \, |ab\rangle . \tag{53}$$

Here $q_{j,|ab\rangle}$ is the measured probability for $|ab\rangle$ after the jth tomography rotation. For any state tomography data, we repeat the same experiment 5000 times to approximate each $q_{j,|ab\rangle}$. We first obtain ρ_{exp} from direct inversion of the experimental data and then perform MLE to find the physical density matrix ρ_m.

The tomographically reconstructed states after preparation and after 9 µs for the three cases of free decay, 4 QQ echo, and AQEC are illustrated in Supplementary Fig. 16. We also plot the decay of the most sensitive off-diagonal matrix element for each logical state (Supplementary Fig. 17) and the fitting to the exponential decays for $|L_0\rangle$ and $|L_1\rangle$ [13]. We use the operator $\tilde{X} = (|gg\rangle + |ff\rangle)(\langle gf| + \langle fg|)/4 + h.c.$ for showing $|L_x\rangle$'s coherence [14]. This operator includes contributions from the four off-diagonal terms in the density matrices that represent the logical phase stability, and the expectation value of $\tilde{X}$ vanishes when logical phase correlations are completely lost.

SUPPLEMENTARY NOTE 9: THEORETICAL CONSTRUCTION OF THE STAR CODE

Supplementary Fig. 18 shows the energy level diagram under the action of different QQ sideband combinations in the fully rotated frame. All nine eigenstates are plotted in the figure in the presence of QQ red sidebands $|ee\rangle \leftrightarrow |gf\rangle$ and $|ee\rangle \leftrightarrow |fg\rangle$ without detunings ((a) top), QQ red sidebands with detunings $\pm\nu_r$ ((a) bottom), QQ blue sidebands $|ee\rangle \leftrightarrow |gg\rangle$ and $|ee\rangle \leftrightarrow |ff\rangle$ without detunings ((b) top), QQ blue sidebands with detunings $\pm\nu_b$ ((b) bottom), and (c) all 4 QQ sidebands without detunings. Here $E_1(\nu) = (\nu + \sqrt{\nu^2 + W^2})/2$ and $E_2(\nu) = (\nu - \sqrt{\nu^2 + W^2})/2$ are the eigenenergies modified by detunings, $k_1(\nu) = (-\nu + \sqrt{2W^2 + \nu^2})/W$ and $k_2(\nu) = (-\nu - \sqrt{2W^2 + \nu^2})/W$ are the coefficients of $|ee\rangle$ components. In Supplementary Fig. 18 (a), the logical state $|L_0\rangle$ (similar for $|L_1\rangle$) is unaffected in the presence of detunings. Without QQ sideband detunings, Supplementary Fig. 18(c) shows that the logical manifold shares the same energy as one stray eigen states (shown in yellow), and that is the reason for adding detunings ν_r and ν_b in Star code. The energy diagram for the full Star code is shown in Section. .

SUPPLEMENTARY NOTE 10: SIMULATION AND ERROR CHANNELS IN THE AQEC

All simulations are carried out in a Hamiltonian of dimension $3 \times 3 \times 2 \times 2$ (two levels for each resonator). We first simulate the theoretical lifetime improvement with only photon-loss error in the static frame ($\tilde{H}_{\text{static}}$ shown in Section.), and the results are shown in Supplementary Fig. 19. All simulated data show improvements beyond the break-even point, even with only 10 µs T_1^{ge} and modest rate requirements for QQ and QR sidebands. The logical coherence limits come from the double photon-loss event and off-resonant population to other stray states from the spectrum crowding (see Ref. [11]). A logical error happens when a second photon decays (from $|e\rangle$ to $|g\rangle$) before getting corrected. $|L_x\rangle$ has a higher lifetime than $|L_j\rangle$ because it is partially protected against double photon loss in a single transmon (See discussion in Section.). Longer physical T_1^{ge}, larger $W_{r/b}$, and Ω_j for a faster error correction rate help increase this limit.

To simulate the real system, several error channels are introduced in the static frame $\tilde{H}_{\text{static}}$ — single photon decay $T_1^{ge,j}$ and $T_1^{ef,j}$, single transmon dephasing T_ϕ^j, single-photon excitation $T_1^{\uparrow}$, resonator photon population n_{res} and extra correlated dephasing $T_\phi^{|ff\rangle}$ at $|ff\rangle$ level. Since only ZZ_{ff1} and ZZ_{ff2} have effects on the logical state, we model the ZZs by directly adding energy shifts to $|fe\rangle$ and $|ef\rangle$, so that all logical basis still share the same energy and remain static in the frame. In the presence of external drives, the parameters will be different from the free decay case. We use experimentally measured ZZ_{ff1} and ZZ_{ff2} values in the simulation. The full master equation is solved in QuTip,

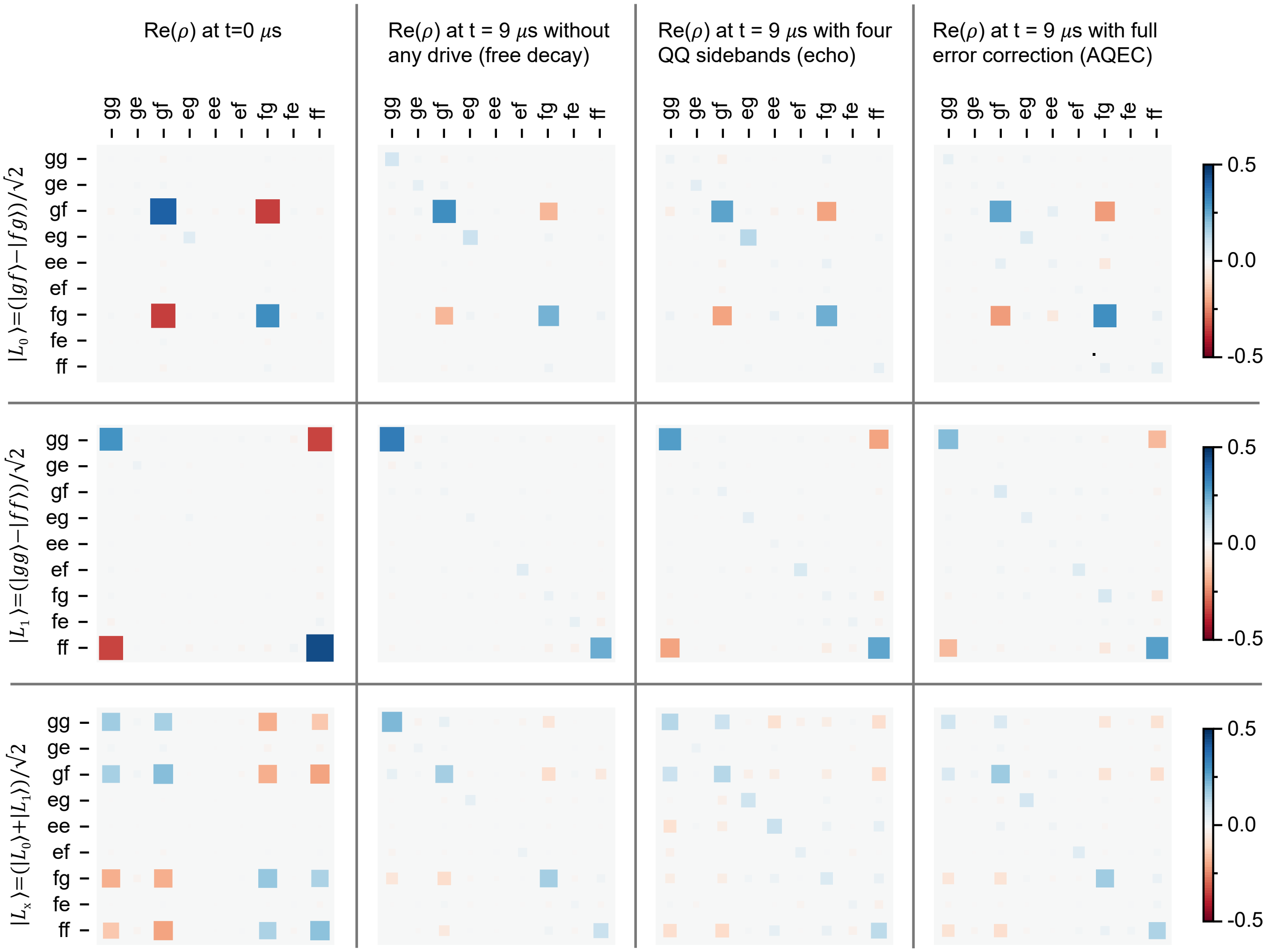

Supplementary Figure 16. Evolution of the logical states under different conditions. Panels from top to bottom correspond to the logical state $|L_0\rangle$, $|L_1\rangle$ and $|L_x\rangle$. The real part of the density matrices are plotted as the imaginary components are small after phase rotation. The left column shows the initial states. Improvements in the coherence can be seen for the echo case when compared to free decay. With the full Star code protocol, further improvements are observed.

$$
\begin{aligned}
\frac{\partial \rho(t)}{\partial t} &= -i\,[H_{\text{full}}, \rho(t)] \\
&+ (\sum_{j=1,2} (\frac{1}{T_1^{ge,j}} D_j[|g\rangle\langle e|] + \frac{1}{T_1^{ef,j}} D_j[|e\rangle\langle f|] \\
&+ \frac{1}{T_1^{\uparrow}} D_j[|e\rangle\langle g|] + \frac{2}{T_1^{\uparrow}} D_j[|f\rangle\langle e|] \\
&+ \frac{1}{T_\phi^{j}} D_j[|e\rangle\langle e|] + \frac{4}{T_\phi^{j}} D_j[|f\rangle\langle f|] \\
&+ \kappa_j n_{\text{res}} D[a_{rj}^{\dagger}] + (1+n_{\text{res}})\kappa_j D[a_{rj}]) \\
&+ \frac{1}{T_\phi^{|ff\rangle}} D_{12}[|ff\rangle\langle ff|])\rho(t).
\end{aligned} \tag{54}
$$

Here we define

$$
\begin{aligned}
H_{\text{full}} &= \tilde{H}_{\text{static}} + \sum_{j=1,2} \chi_j n_{qj} n_{rj} \\
&\quad + (ZZ_{ff1}\,|fe\rangle\langle fe| + ZZ_{ff2}\,|ef\rangle\langle ef|) \otimes I_4,
\end{aligned} \tag{55}
$$

$$D[A]\rho = A\rho A^{\dagger} - \frac{1}{2}\left(A^{\dagger}A\rho + \rho A^{\dagger}A\right), \tag{56}$$

$$D_1[A] = D[A \otimes I_3 \otimes I_4], \tag{57}$$

$$D_2[A] = D[I_3 \otimes A \otimes I_4], \tag{58}$$

$$D_{12}[A] = D[A \otimes I_4]. \tag{59}$$

Since transmons' anharmonicities are much larger than the transmon decay rate, each level's decay and dephasing are phase-independent. The system's full density matrix $\rho(t)$ is calculated and used to extract the coherence time and correctable error rate. Supplementary Table 6

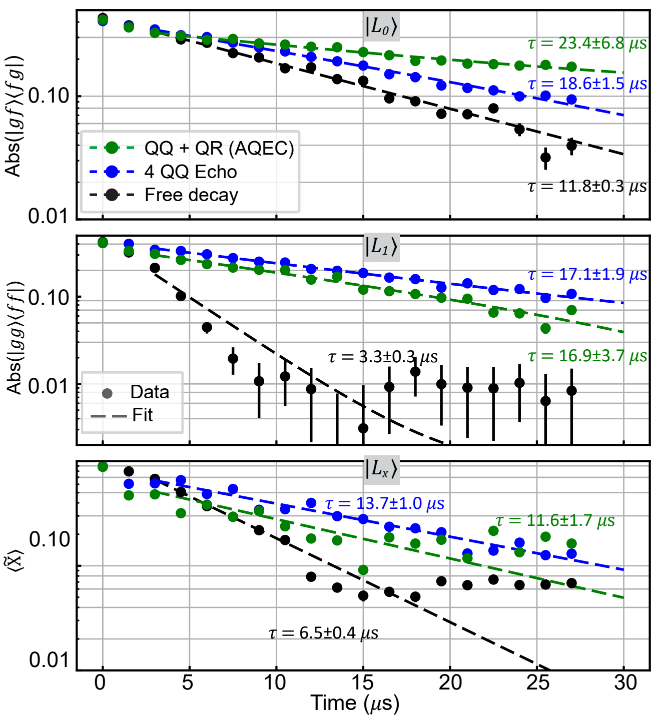

Supplementary Figure 17. Black, blue, and green circles are experimentally obtained expectation values for the relevant operators representing coherence at a given time. The expectation values are extracted from the tomographic reconstruction of states with 5000 repeated measurements. Error bars (one standard deviation) are calculated using the Tomographer package [15].

Simulation parameters	Free decay	4 QQ echo	AQEC
Q_1 $T_1^{ge,1}(\mu s)$	18.0	21.0	21.0
Q_1 $T_1^{ef,1}(\mu s)$	33.0	29.0	23.0
Q_1 $T_\phi^{1}(\mu s)^\dagger$	15.0	23.0	23.0
Q_1 $T_1^{\uparrow}(\mu s)$	∞	∞	600.0
Q_2 $T_1^{ge,2}(\mu s)^*$	8.0	9.0	9.0
Q_2 $T_1^{ef,2}(\mu s)$	33.0	29.0	23.0
Q_2 $T_\phi^{2}(\mu s)^\dagger$	15.0	23.0	23.0
Q_2 $T_1^{\uparrow}(\mu s)$	∞	∞	600.0
$T_\phi^{\lvert ff\rangle}(\mu s)$	4.4	80.0	80.0
κ_1 (MHz)	0.53	0.53	0.53
κ_2 (MHz)	0.48	0.48	0.48
χ_1 (MHz)			−0.2
χ_2 (MHz)			−0.2
$n_{\rm res}$	0.00	0.00	0.03
W_r (MHz)		1.00	1.45
W_b (MHz)		1.70	1.25
ν_r (MHz)		1.50	0.80
ν_b (MHz)		0.00	−0.90
Ω_1 (MHz)			0.39
Ω_2 (MHz)			0.39
ZZ_{ff1} (MHz)			0.6
ZZ_{ff2} (MHz)			2.2

Supplementary Table 6. Parameters used in the master equation simulation. $\{W_{r/b}, \Omega_j, \nu_{r/b}\}$ are extracted through Supplementary Fig. 12 in simulation; ZZ_{ff1} and ZZ_{ff2} are experimentally measured through $|e0\rangle \leftrightarrow |f1\rangle$ on-resonance frequency difference when all sidebands on. All coherence numbers $\{T_1, T_\phi\}$, $n_{\rm res}$, and χ_j are free parameters chosen in the simulation to match the experimental data. The other parameters listed are all obtained from measurements. Irrelevant parameters in each case are not shown in the table and are not included in the simulation.
† Dephasing in the 4 QQ echo and AQEC cases are higher because of the QQ sideband spin-echo improvement.
* Q_2's T_1^{ge} is lower than Q_1's because of the TLSs around the transition frequency. Effects to the codewords performance are minimal as the population on $|e\rangle$ is corrected.

includes all parameters used in the master equation simulation. The final simulation results for the experimental comparison are shown in Supplementary Fig. 20, and the comparison using fidelity metric is shown in Supplementary Fig. 21. For each separate case (free decay, 4 QQ echo, and AQEC), parameters are the same for all logical states $|L_0\rangle$, $|L_1\rangle$, and $|L_x\rangle$. T_ϕ^j is increased in the 4 QQ Echo and AQEC cases because of the echo suppression of $1/f$ noise.

Supplementary Table 7 shows the lifetime limitations from different error channels in the AQEC case. In the ideal implementation, we include only the single photon decay and QR couplings χ_j in the simulation. The transmon photon excitation is enhanced when all sidebands are turned on. However, excitation error on $|L_x\rangle$ is partially correctable under three-level approximation, and thus $|L_x\rangle$ is more insensitive to it compared to $|L_{0/1}\rangle$. Resonator photon excitation happens from the heating effect when QR sidebands are on. Larger cavity photon number $n_{\rm res}$ will dephase all logical states and is one of the dominant error sources in our system. With higher resonator frequencies or an extra coupler between transmon and resonator, $n_{\rm res}$ can be reduced under the same QR rate Ω_j. The next two dominant error channels are cross-Kerr of between the transmons and QR frequency mismatch. ZZ_{ff1} and ZZ_{ff2} will dephase logical superposition states, as discussed in Supplementary Note 4 (but has no effect on individual logical states). QR frequency mismatch is unavoidable in the presence of ZZ. In the experiment, we apply on-resonance $|e0\rangle \leftrightarrow |f1\rangle$ drive for $|L_0\rangle$ (corresponding the partner transmon being in $|g\rangle$). Consequently, for $|L_1\rangle$ the QR sidebands become detuned by ZZ_{ff1} and ZZ_{ff2} (corresponding to the partner being in $|f\rangle$) and effectively perform slower error correction. The QQ sideband frequency mismatch comes from a modest upper bound of the system's frequency drift (around 10 kHz). This is not comparable to the W and has no significant influence on the logical states. Other dephasing noise sources include $1/f$ noise, white noise, and $|ff\rangle$'s correlated dephasing. Among those three, the white noise affects AQEC performance as it has a constant noise spectrum that cannot be suppressed through the spin echo. Star code protocol is also insensitive to small sideband amplitude drifts. The phase

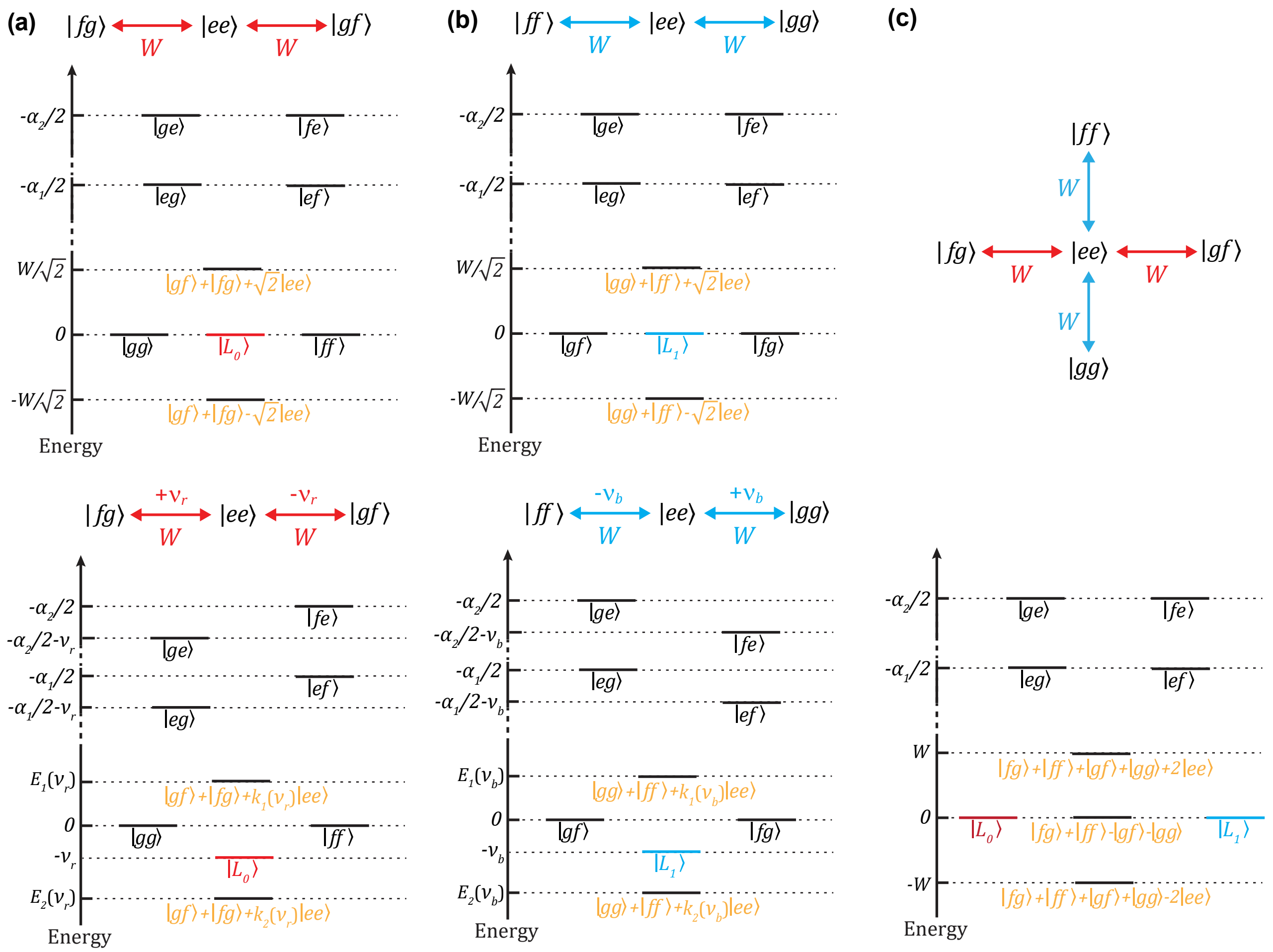

Supplementary Figure 18. Step-by-step construction of the Star code. Two qutrits' energy level diagrams under QQ red sidebands only ((a) top and bottom), QQ blue sidebands only ((b) top and bottom), and all QQ sidebands without detunings (c) are shown in the plot. For representation simplicity, the eigenstates are not normalized. Definitions of E_i and k_i are shown in the text.

between logical states is defined by different sideband pairs, and amplitude drifts have to be comparable to W to change the logical states. Further, when all sidebands are on, both transmons' physical T_1 are shortened, which slightly reduces the performance. Other insignificant error sources include leakage to higher transmon energy levels ($|\alpha_j| \gg W$) and population in the coupler mode ($\omega_c \gg \omega_j$). Those are not considered in the simulation as the transition frequency is far away.

SUPPLEMENTARY NOTE 11: DEVICE FABRICATION AND MEASUREMENT SETUP

The substrate for the device is a 430 μm thick C-plane sapphire wafer annealed at 1200°C for 2 hours. The ground plane uses 200 nm thick Tantalum film sputtered at 800°C. Large patterns, except Josephson junctions, were made through optical lithography and 20-second wet-etching in Transene Tantalum etchant 111. AZ 1518 was spin-coated as the positive photoresist, and a Heidelberg MLA 150 Direct Writer was used for the photolithography. The junction mask was fabricated with a Raith EBPG5000 Plus E-Beam Writer on a bi-layer resist (MMA EL11-950 PMMA A7). Transmon and coupler's Josephson junctions are Dolan bridge type. The mask was evaporated in a Plassys electron-beam evaporator with double-angle evaporation ($\pm 23^o$). The wafer was diced into 7×7 mm^2 chips and lifted off. After measuring the test junctions' resistances, the device was mounted on a printed circuit board, wire-bonded, packaged inside a μ-metal shielded sample can, and installed inside a dilution fridge.

Supplementary Fig. 22 shows the room and cryogenic temperature measurement chain. The device is mounted on the mixing chamber plate of the dilution fridge with a 15 mK base temperature. A Tektronix 5014C AWG (1.2 GSa/s) acts as the master trigger for all other equip-

Error channels limit	$\lvert L_0\rangle$	$\lvert L_1\rangle$	$\lvert L_y\rangle^{\ddagger}$	$\lvert L_x\rangle$
Ideal implementation*	95 µs		95 µs	160 µs
Transmon photon excitation	∼ 360 µs		360 µs	∼ 3 ms
n_{res} dephasing†	∼ 55 µs		∼ 40 µs	∼ 40 µs
Other dephasing noise†	∼ 50 µs		∼ 35 µs	∼ 35 µs
Transmon ZZ dephasing	∞		∼ 30 µs	∼ 30 µs
QR frequency mismatch	> 10 ms	∼ 45 µs		
QQ frequency mismatch	> 10 ms		> 10 ms	∼ 1.5 ms
QQ rate mismatch	> 10 ms		> 10 ms	∼ 4 ms
Reduced physical T_1	∼ 330 µs		∼ 330 µs	∼ 400 µs
Total predicted lifetime	∼ 18.2 µs	∼ 13.0 µs	∼ 10.0 µs	∼ 10.3 µs
Experimental lifetime	28.1 ± 10.0 µs	16.3 ± 2.6 µs		5.2 ± 2.0 µs

Supplementary Table 7. Various decoherence channels for the logical qubit. Ideal implementation represents logical states' lifetime with QR coupling and only T_1 error. Each limit is extracted using the simulation through lifetime difference after adding relevant error channels. The average photon number in the resonator (n_{res}) increases during external drives and dephases transmons through photon shot noise. Other dephasing noise include $1/f$ noise, white noise, correlated dephasing noise, and any other noise source. The total effect is represented with T_ϕ^j in the simulation. ZZ between transmons introduces a large mismatch in QR frequency for $\lvert L_1\rangle$ and $\lvert L_x\rangle$, and the effect is combined with ZZ dephasing for $\lvert L_x\rangle$ case. The drifts in sideband amplitudes frequencies are less than 5% and 20 kHz, and those limits are in the order of ms.
* Ideal implementation includes QR couplings χ_j but no QQ ZZ couplings.
† n_{res} and T_ϕ^j are determined through simulation-experiment matching.
‡ $\lvert L_y\rangle$'s lifetime is estimated through simulation, based on $\lvert L_x\rangle$'s experimental error channels.

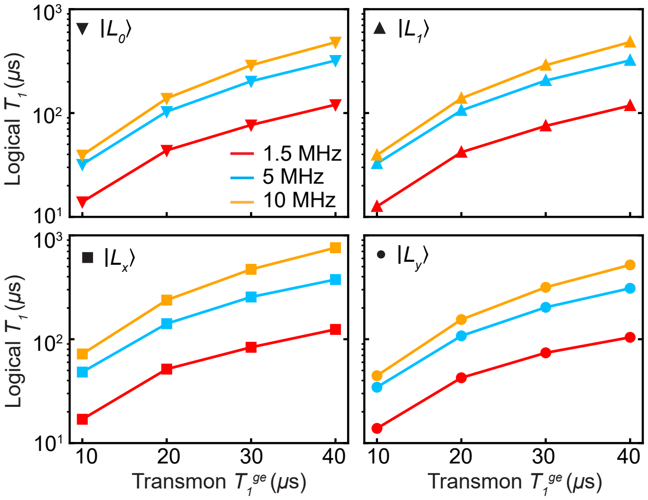

Supplementary Figure 19. Theoretical logical lifetime in the rotating frame. The logical lifetime increases as a function of physical T_1. A larger QQ sideband rate also provides higher logical qubit coherence. Parameter used for simulation are $\{W = 10, 5\,\mathrm{MHz}, \Omega_j = 1.0\,\mathrm{MHz}, \nu_r = -\nu_b = \frac{W}{2}\}$, $\{W = 1.5\,\mathrm{MHz}, \Omega_j = 0.4\,\mathrm{MHz},\ \nu_r = -\nu_b = 0.85\,\mathrm{MHz}\}$ and $\kappa = 0.5\,\mathrm{MHz}$.

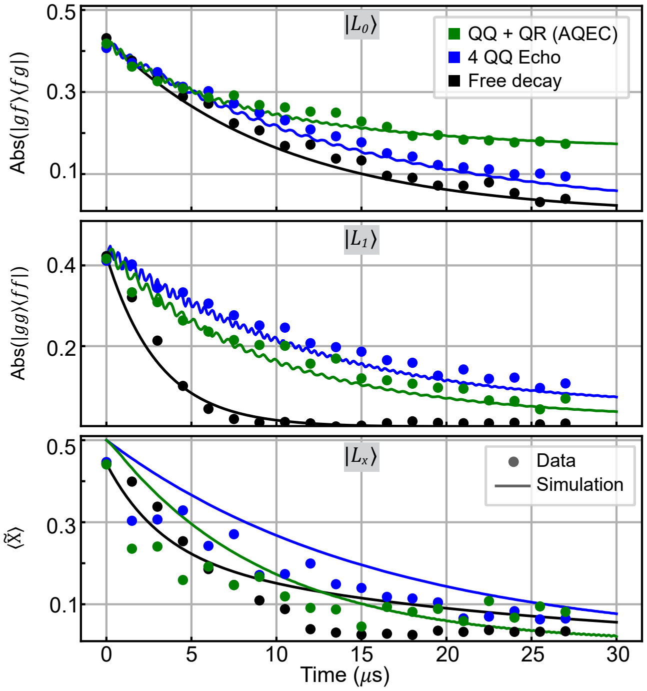

Supplementary Figure 20. Simulation of the Star code performance in the static frame with the experimental data. Error bars (one standard deviation) are smaller than the marker size.

ment. The readout pulses are generated through two CW tones from RF sources (PSG-E8257D), and modulated by AWG 5014C. The qubit input pulses are generated through another 4-channel AWG (Keysight M8195 65 GSa/s, 16 GSa/s per channel). The qubit and readout signals are combined and sent through lines In_1 and In_2 into the dilution fridge. Three DC sources (Yokogawa GS200) are used to bias the DC flux of the coupler and two Josephson Parametric Amplifiers (JPA). The red and blue QQ RF flux drives and two direct QR charge drives are synthesized through the same 4-channel AWG. Inside the fridge, at the 4K plate, all input lines have 20-dB attenuators. At the base plate, In_1 and In_2 lines have 10-dB attenuators, followed by a strong Eccosorb® provid-

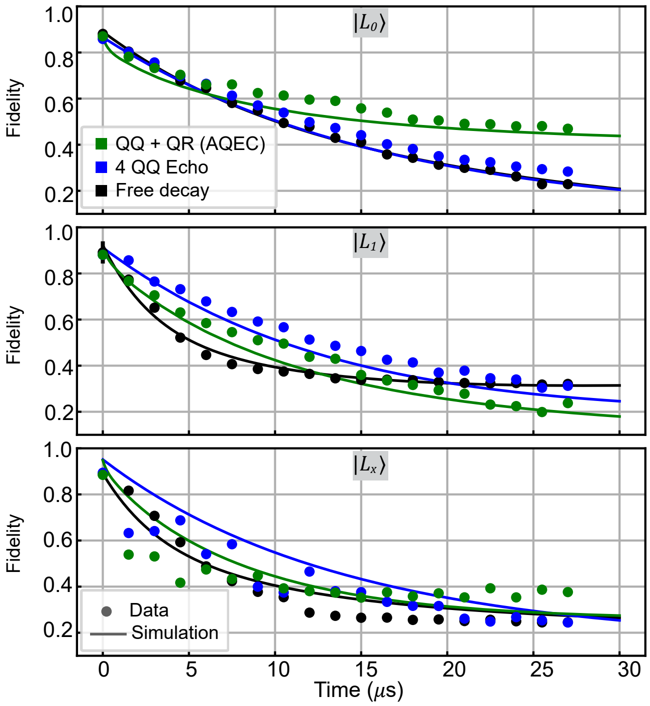

Supplementary Figure 21. Simulation of the Star code performance in the static frame with the experimental data using fidelity metric. Error bars (one standard deviation) are smaller than the marker size.

ing 20-dB attenuation at 4 GHz. Charge_1 and Charge_2 lines have 20-dB attenuators, followed by strong Eccosorb providing 20-dB attenuation at 4 GHz, and a bandpass filter with passband $3.9 - 4.8$ GHz. The DC Flux line has a low pass filter (DC $-$ 1.9 MHz) and a weak Eccosorb (1-dB attenuation at 4 GHz). The red-frequency RF flux line passes through a weak Eccosorb first, followed by a high pass filter (cut off at 200 MHz) and a low pass filter (cut off at 2 GHz). The blue-frequency RF flux line passes through a weak Eccosorb first, followed by a high pass filter (cut off at 6 GHz). The two RF flux lines and the DC flux line are combined and pass through a Step Impedance Purcell Filter (SIPF) with a stop band $2.5 - 5.5$ GHz. The two output signals go through three circulators, then each amplified by a JPA with 15-dB gain, followed by a low pass filter (cut off at 8 GHz), two circulators, a DC block, and amplified with one LNF High-Electron-Mobility Transistor (HEMT) amplifier. The output signals are further amplified at room temperature, then demodulated, filtered with a low pass filter (DC $-$250 MHz), and amplified again using the SRS Preamplifier. The final signal is digitized with Alazar ATS 9870 (1 GSa/s) and analyzed in a computer.

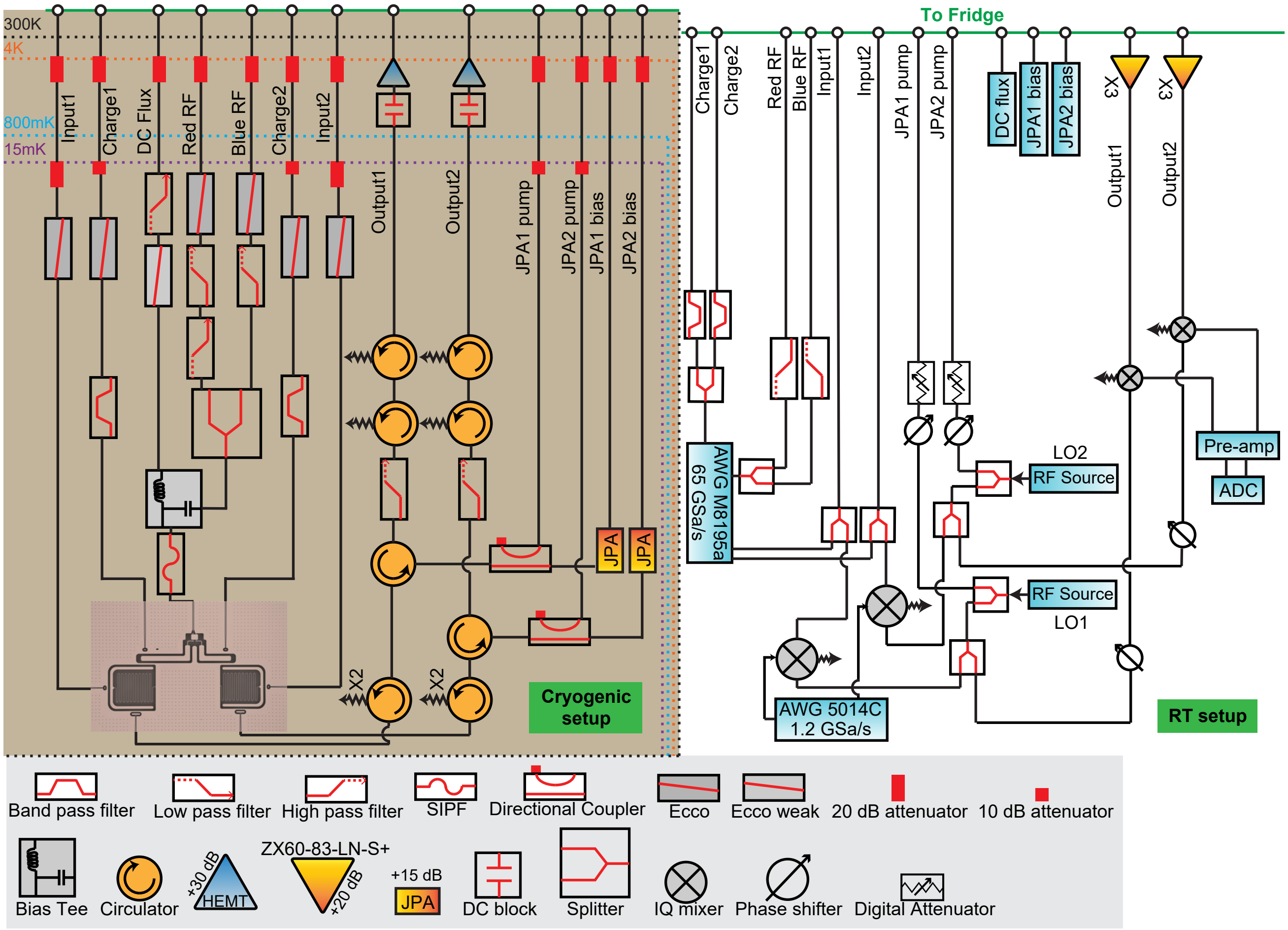

Supplementary Figure 22. Detailed cryogenic and room temperature measurement setup.

[1] S. P. Chitta, T. Zhao, Z. Huang, I. Mondragon-Shem, and J. Koch, Computer-aided quantization and numerical analysis of superconducting circuits (2022).

[2] Y. Lu, S. Chakram, N. Leung, N. Earnest, R. K. Naik, Z. Huang, P. Groszkowski, E. Kapit, J. Koch, and D. I. Schuster, Universal stabilization of a parametrically coupled qubit, Phys. Rev. Lett. **119**, 150502 (2017).

[3] M. Roth, M. Ganzhorn, N. Moll, S. Filipp, G. Salis, and S. Schmidt, Analysis of a parametrically driven exchange-type gate and a two-photon excitation gate between superconducting qubits, Phys. Rev. A **96**, 062323 (2017).

[4] A. Wallraff, D. I. Schuster, A. Blais, J. M. Gambetta, J. Schreier, L. Frunzio, M. H. Devoret, S. M. Girvin, and R. J. Schoelkopf, Sideband transitions and two-tone spectroscopy of a superconducting qubit strongly coupled to an on-chip cavity, Phys. Rev. Lett. **99**, 050501 (2007).

[5] J. Chu, D. Li, X. Yang, S. Song, Z. Han, Z. Yang, Y. Dong, W. Zheng, Z. Wang, X. Yu, D. Lan, X. Tan, and Y. Yu, Realization of superadiabatic two-qubit gates using parametric modulation in superconducting circuits, Phys. Rev. Applied **13**, 064012 (2020).

[6] M. Ganzhorn, G. Salis, D. J. Egger, A. Fuhrer, M. Mergenthaler, C. Müller, P. Müller, S. Paredes, M. Pechal, M. Werninghaus, and S. Filipp, Benchmarking the noise sensitivity of different parametric two-qubit gates in a single superconducting quantum computing platform, Phys. Rev. Research **2**, 033447 (2020).

[7] E. A. Sete, N. Didier, A. Q. Chen, S. Kulshreshtha, R. Manenti, and S. Poletto, Parametric-resonance entangling gates with a tunable coupler, Phys. Rev. Applied **16**, 024050 (2021).

[8] T. Brown, E. Doucet, D. Ristè, G. Ribeill, K. Cicak, J. Aumentado, R. Simmonds, L. Govia, A. Kamal, and L. Ranzani, Trade off-free entanglement stabilization in a superconducting qutrit-qubit system, Nature Communications **13**, 3994 (2022).

[9] J. C. Gallop and B. W. Petley, SQUIDs and their applications, Journal of Physics E: Scientific Instruments **9**, 417 (1976).

[10] T. Roy, Z. Li, E. Kapit, and D. I. Schuster, Realization of two-qutrit quantum algorithms on a programmable superconducting processor, Preprint at `https://arxiv.org/abs/2211.06523` (2022).

[11] Z. Li, T. Roy, D. R. Pérez, D. I. Schuster, and E. Kapit, Hardware efficient autonomous error correction with linear couplers in superconducting circuits, Preprint at `https://arxiv.org/abs/2303.01110` (2023).

[12] R. Bianchetti, S. Filipp, M. Baur, J. M. Fink, C. Lang, L. Steffen, M. Boissonneault, A. Blais, and A. Wallraff, Control and tomography of a three level superconducting artificial atom, Phys. Rev. Lett. **105**, 223601 (2010).

[13] D. Rodríguez Pérez, Quantum error mitigation and autonomous correction using dissipative engineering and coupling techniques, ProQuest Dissertations and Theses , 147 (2021).

[14] E. Kapit, Error-transparent quantum gates for small logical qubit architectures, Phys. Rev. Lett. **120**, 050503 (2018).

[15] P. Faist and R. Renner, Practical and reliable error bars in quantum tomography, Phys. Rev. Lett. **117**, 010404 (2016).
